# Dmp1 Lineage Cells Contribute Significantly to Periosteal Lamellar Bone Formation Induced by Mechanical Loading But Are Depleted from the Bone Surface During Rapid Bone Formation

**DOI:** 10.1002/jbm4.10593

**Published:** 2022-01-04

**Authors:** Taylor L. Harris, Matthew J. Silva

**Affiliations:** ^1^ Department of Orthopaedic Surgery and Musculoskeletal Research Center Washington University School of Medicine St. Louis MO USA; ^2^ Department of Biomedical Engineering Washington University St. Louis MO USA

**Keywords:** GENETIC ANIMAL MODELS, ANIMAL MODELS, OSTEOBLASTS, CELLS OF BONE, MATRIX MINERALIZATION, BONE MATRIX

## Abstract

Previous work has shown that osteoprogenitor cells (Prx1+) and pre‐osteoblasts (Osx+) contribute to mechanical loading‐induced bone formation. However, the role of mature Dmp1‐expressing osteoblasts has not been reported. In this study we assessed the contribution of osteoblast lineage cells to bone formation at an early time point following mechanical loading (day 8 from onset of loading). We labeled Osx‐expressing and Dmp1‐expressing cells in inducible Osx and Dmp1 reporter mice (iOsx‐Ai9, iDmp1‐Ai9), respectively, 3 weeks before loading. Mice were then loaded daily for 5 days (days 1–5) and were dosed with 5‐ethynyl‐2′‐deoxyuridine (EdU) in their drinking water until euthanasia on day 8. Mice were loaded to lamellar and woven bone inducing stimulation (−7 N/1400 με, −10 N/2000 με) to assess differences in these processes. We found varied responses in males and females to the loading stimuli, inducing modest lamellar (females, −7 N), moderate lamellar (males, −10 N), and robust woven (females, −10 N) bone. Overall, we found that preexisting (ie, lineage positive) Osx‐expressing and Dmp1‐expressing cells contribute largely to the bone formation response, especially during modest bone formation, while our results stuggest that other (non‐lineage–positive) cells support the sustained bone formation response during rapid bone formation. With moderate or robust levels of bone formation, a decrease in preexisting Osx‐expressing and Dmp1‐expressing cells at the bone surface occurred, with a near depletion of Dmp1‐expressing cells from the surface in female mice loaded to −10 N (from 52% to 11%). These cells appeared to be replaced by lineage‐negative cells from the periosteum. We also found a dose response in proliferation, with 17% to 18% of bone surface cells arising via proliferation in modest lamellar, 38% to 53% in moderate lamellar, and 59% to 81% in robust woven bone formation. In summary, our results show predominant contributions by preexisting Osx and Dmp1 lineage cells to loading‐induced lamellar bone formation, whereas recruitment of earlier osteoprogenitors and increased cell proliferation support robust woven bone formation. © 2021 The Authors. *JBMR Plus* published by Wiley Periodicals LLC on behalf of American Society for Bone and Mineral Research.

## Introduction

There are two classes of US Food and Drug Administration (FDA)‐approved anabolic therapies to treat advanced bone loss: parathyroid hormone and its analogues, and sclerostin antibody.^(^ ^1^ ^)^ But with these, there are certain contraindications for use and limited periods of effectiveness. A better understanding of the cellular mechanisms for initiating and maintaining bone formation may elucidate new treatment strategies for bone loss.

Mechanical loading of rodents has led to a better understanding of key molecular and cellular events leading to bone formation in vivo.^(^ ^2^ ^)^ Earlier studies identified proliferation and activation of bone lining cells as cell sources contributing to bone formation,^(^ ^3^, ^4^, ^5^ ^)^ although these studies were confounded by either invasive methods or direct loading contacts on the periosteum. Uniaxial compressive limb loading in mice has alleviated these problems and become the prevailing tool for understanding physiological bone formation in vivo.^(^ ^6^ ^)^ With this approach, the importance of increased proliferation and activation has been reported,^(^ ^7^, ^8^ ^)^ specifically in Osx‐lineage cells at the bone surface^(^ ^7^ ^)^ and in osteochondroprogenitors (Prx1^+^) cells.^(^ ^9^, ^10^ ^)^

The differentiation stage of cells directly contributing to bone formation, and the role of proliferation for the initiation and sustainment of bone formation have not been fully defined. Cabahug‐Zuckerman and colleagues^(^ ^9^ ^)^ measured the presence of Prx1, Sca‐1, and Ki67 markers within the entire periosteum, showing an increase in Prx1^+^Sca1^+/−^ proliferation on days 2 and 4, but not on day 6, following the onset of tibial loading to induce lamellar bone formation. This result is useful to understand what is occurring overall in the periosteum, but the types of cells identified could not be attributed directly to formation of bone at the surface. Moore and colleagues^(^ ^10^ ^)^ utilized an inducible Prx1 mouse to knock out osteoprogenitors. Interestingly, this technique attenuated the loading response in ulnae but did not entirely abrogate lamellar bone formation 15 days following the start of loading. From these studies, it is clear that progenitor cells proliferate in the periosteum following loading at early time points in lamellar bone formation, and osteoprogenitors are necessary to sustain lamellar bone formation at later time points following loading.

Using Osx‐Cre‐ERT2;Ai9 reporter mice transiently induced with tamoxifen 3 weeks prior to loading, Zannit and Silva^(^ ^7^ ^)^ showed that >95% of the lamellar bone forming surface was covered by preexisting Osx‐lineage cells after day 5 of loading, which is near the time when mineralized bone first forms.^(^ ^11^ ^)^ However, it is unclear whether bone formation was generated by preosteoblasts, or cells later in lineage, because this reporter mouse marks cells at many stages of osteoblast differentiation. The Dmp1‐Cre‐ERT2;Ai9 reporter mouse labels the lineage from mature osteoblasts through osteocytes.^(^ ^12^, ^13^, ^14^ ^)^ In the current study, we therefore used both inducible Osx and Dmp1 reporter mice to better distinguish the role of preosteoblasts from mature osteoblasts. Further, to investigate whether Osx‐lineage cells continue to sustain bone formation past day 5, we sought to evaluate bone formation at day 8 following loading. We suspected that the preexisting Osx and Dmp1 lineage cells are important for initiation of bone formation, and that recruitment and differentiation of osteoprogenitors plays a greater role in sustaining the bone formation response at the later time point. Finally, these aspects of bone formation have not been investigated in slower lamellar versus more rapid woven bone formation, both of which can be induced in a strain‐dependent manner by in vivo loading. Thus, we chose two force levels to induce lamellar and woven bone to examine differences in these processes.

Zannit and Silva^(^ ^7^ ^)^ also reported some Osx‐lineage cells embedded in newly formed matrix, although these were not quantified. Recent evidence suggests that osteocytes actively embed into their matrix beginning with the simultaneous occurrence of Dmp1 expression and mineralization.^(^ ^15^, ^16^ ^)^ Thus, we used our model to investigate if preexisting Dmp1‐expressing cells are more likely to embed compared to cells earlier in the osteoblast lineage.

Our goal herein was to determine the contribution of osteoblast lineage cells to bone formation at day 8 following induction of either lamellar or woven bone formation in young‐adult mice. We utilized Osx and Dmp1 reporter mice to mark cells at two stages of differentiation and a thymidine analog to detect proliferation occurring throughout the experiment. Finally, we quantified cells in three distinct regions: directly on the bone surface, in the adjacent periosteum, and within newly formed bone.

## Materials and Methods

### Mice

**Fig. 1:**
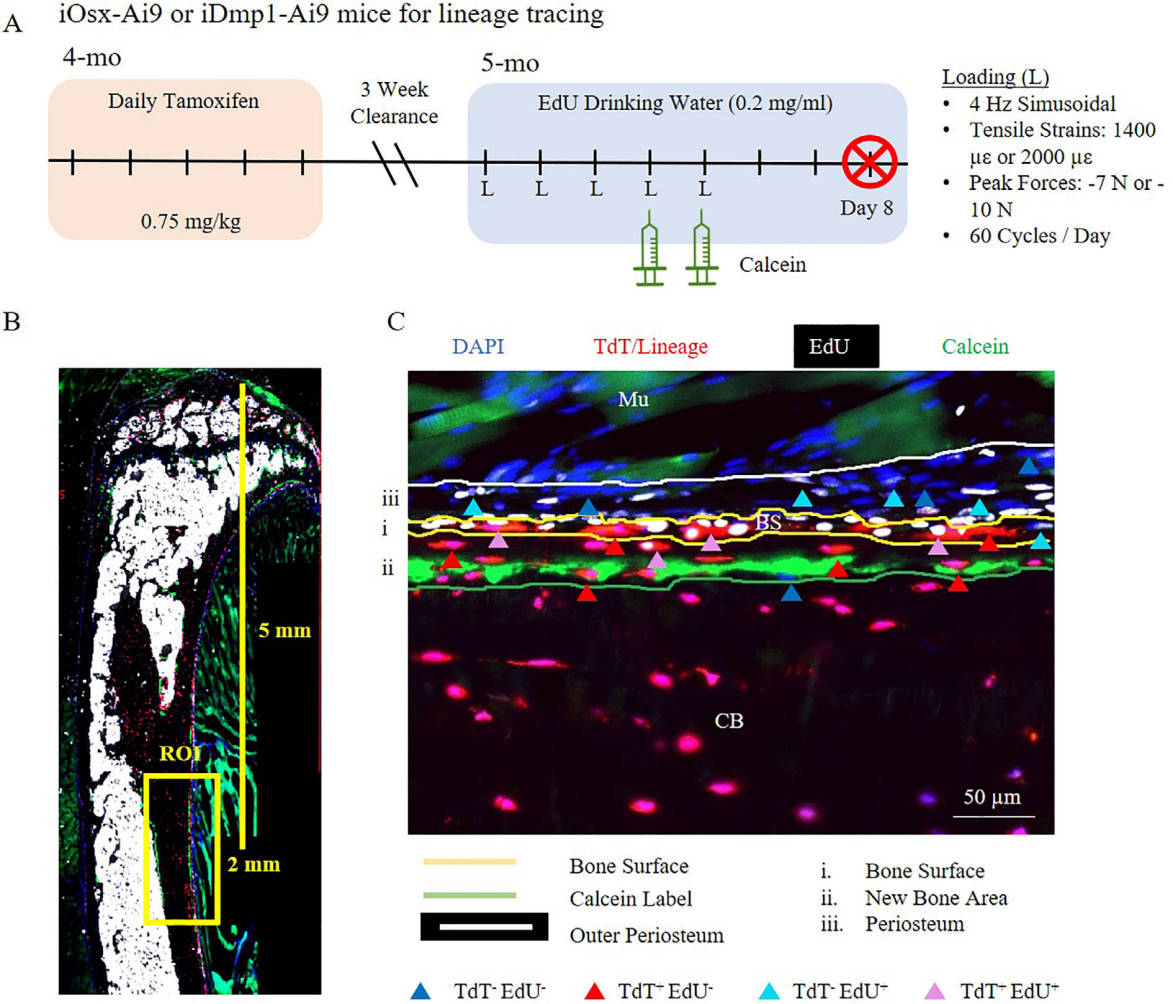
Methods for ROI selection and quantification of cell type. (*A*) Timeline for lineage tracing outcomes. (*B*) A 2‐mm ROI was selected 5 mm distal to the tibial plateau. (*C*) Regions were divided into bone surface (i), new bone area (ii), and periosteum (iii). Cells were counted as TdT^−^ EdU^−^ (dark blue), TdT^+^ EdU^−^ (dark red), TdT^−^ EdU^+^ (light blue), or TdT^+^ EdU^+^ (pink). BS = bone surface; CB = cortical bone; Mu = muscle; ROI = region of interest.

Transgenic Osx Cre‐ERT2 (iOsx)^(^ ^17^ ^)^ mice were provided by Henry Kronenberg (Harvard University) for lineage‐tracing experiments. Mice were bred to Ai9 reporter mice that express the TdTomato (TdT) fluorescent protein under the presence of Cre in the nucleus (C57Bl/6J background; The Jackson Laboratory, Bar Harbor, ME, USA; #007909)^(^ ^18^ ^)^ to produce iOsx‐Ai9 reporter mice. The Osx‐Cre‐ERT2^+/−^;Ai9^+/− (or +/+)^ offspring were used for conditionally labeling Osx‐expressing cells upon administration of tamoxifen (TAM; MilliporeSigma, Burlington, MA, USA). Dmp1 Cre‐ERT2 (iDmp1) mice^(^ ^19^ ^)^ were provided by Paola Pajevic (Boston University) for lineage tracing of osteoblast cells at a later stage in differentiation. This inducible Dmp1 mouse utilizes the 10‐kilobase (kb) fragment of Dmp1, which is known to be expressed by osteocytes and some mature osteoblasts.^(^ ^12^, ^19^, ^20^ ^)^ Similar breeding schemes were utilized to generate Dmp1‐Cre‐ERT2^+/−^;Ai9^+/− (or +/+)^ inducible reporter mice (iDmp1‐Ai9). Both Osx and Dmp1 Cre mice had been backcrossed onto C57BL/6 prior to receiving and were maintained on this background. Cre^−^;Ai9^+/− (or +/+)^ mice were utilized to control for any possible Cre effects. Mice were group housed by gender in standard 12‐hour light/dark cycle conditions with temperature and humidity controlled between 20°C and 23°C and 30% to 70%, respectively, and fed chow *ad libitum* (Purina, St. Louis, MO, USA; 5053). At approximately 4 months of age, mice were administered 75 mg/kg of TAM daily for 5 days to pulse‐label lineage cells, followed by a 3‐week clearance period to diminish any TAM effects (Fig. 1*A*). Because TAM effects on bone formation have been reported,^(^ ^21^, ^22^ ^)^ and due to leakiness of the Cre transgene under certain promoters,^(^ ^12^, ^13^, ^20^ ^)^ TAM was withheld from a subset of mice (Cre^+^;Ai9^+/− (or +/+)^) as a control. Institutional Animal Care and Use Committee (IACUC) approval was obtained from Washington University before conducting animal experiments.

### Strain gauging

At 5‐months of age, 27 mice were used for postmortem strain gauge analysis (15 iOsx‐Ai9; 12 iDmp1‐Ai9) to estimate the peak forces for loading to generate anteromedial (tensile) strains of 1400 με and 2000 με. These strains correspond to approximately −2200 με and −3200 με peak strains at the posterolateral surface (compressive), previously shown to stimulate lamellar and woven bone, respectively, in C57Bl/6 female mice.^(^ ^23^ ^)^ Using methods previously described,^(^ ^24^ ^)^ strain gages (FLK‐1‐11‐1LJC; Tokyo Measuring Instruments Lab, Tokyo, Japan) were applied to the tensile surface of the tibia, and the tibia was positioned in the same fixtures and subjected to the same loading waveform used for in vivo loading (described below, 'In vivo mechanical loading'). Cyclic loads with peak forces ranging from −2 N to −8 N were applied using a materials‐testing machine (DynaMight 8841; Instron, Inc., Grove City, PA, USA). The recorded strains were used to determine the force‐strain relationship and to predict the peak forces for generating the desired strains. Cre^+^ (*n* = 4 iOsx‐Ai9; *n* = 3 iDmp1‐Ai9) and Cre^−^ (*n* = 7 iOsx‐Ai9; *n* = 6 iDmp1‐Ai9) mice were given tamoxifen, whereas a subset did not receive tamoxifen (*n* = 4 iOsx‐Ai9, *n* = 3 iDmp1‐Ai9). Because no trends due to Cre status or tamoxifen administration were noticeable, Cre^+^ and Cre^−^ mice were grouped regardless of tamoxifen administration.

Strain gauging analysis showed that −7 and −10 N peak forces would engender target strains expected to induce lamellar and woven bone formation, respectively (Fig. S1). Overall, similar strains were produced at the chosen peak forces across mouse strain (iOsx‐Ai9, iDmp1‐Ai9) and between sexes. However, slightly higher strains were induced by these forces in female iOsx‐Ai9 mice (180–260 με more than iOsx‐Ai9 males).

### In vivo mechanical loading

At 5 months of age, the chosen peak forces to stimulate lamellar and woven bone formation were applied via force‐controlled cyclic loading (Electropulse 1000; Instron) to the right hindlimb. Left limbs from the same mice served as contralateral non‐loaded controls. Under anesthesia (1%–3% isoflurane), the tibia of each mouse was positioned between two fixtures holding the knee and ankle in place so that a uniaxial force could be applied to the tibia. A −0.5‐N preload was used to keep the limb in place during loading. Sixty (60) cycles of a 4‐Hz sinusoidal waveform were applied daily for 5 days. Buprenorphine (0.1 mg/kg) was injected subcutaneously or intraperitoneally (days 4 and 5 only) after each loading bout for pain management. Mice were allowed free cage activity following loading. From the start of loading until euthanasia, mice were given a click chemistry thymidine analog (5‐ethynyl‐2′‐deoxyuridine [EdU]; Thermo Fisher Scientific, Waltham, MA, USA; E10415) in their drinking water (0.2 mg/mL). Mice were group‐housed by sex, but controls without TAM were separated from others. Intraperitoneal (ip) injections of calcein green (10 mg/kg; MilliporeSigma) were delivered on days 4 and 5 after loading; two closely spaced doses were given to increase efficiency for marking a single‐labeled surface. Mice were allowed normal activity for 2 days after the last loading event and euthanized on the third day (day 8). Mice were euthanized by CO_2_ asphyxiation.

### Histology

Non‐loaded and loaded tibias were dissected for lineage tracing. Muscle was trimmed without disturbing periosteum. Bones were fixed in 4% paraformaldehyde for 24 hours, washed in PBS 3×, soaked in 30% sucrose for 2 days and Tissue‐Tek optimal cutting temperature compound (OCT; VWR, Leicestershire, UK) for 1 day. Bones were embedded in OCT using a freezing plate and sections were cut longitudinally on a cryostat at 6 μm thickness using HP35 Ultra HP MicroBlades (Thermo Fisher Scientific; 31‐537‐35) and Kawamoto film (Section‐Lab Co. Ltd., Hiroshima, Japan). Sections were mounted to slides with 0.5% chitosan (MilliporeSigma; 419419) in 0.25% acetic acid, and allowed to wick and dry for 2 days before staining.

EdU staining was performed (Click It EdU Cell Proliferation Kit for Imaging, Alexa Fluor 647 dye; Thermo Fisher Scientific; C10340) according to the user manual instructions. 4,6‐Diamidino‐2‐phenylindole (DAPI) counterstain was performed (MilliporeSigma; D9452) for 10 minutes following EdU staining. Sections were coverslipped using Fluoromount‐G mounting media (Invitrogen, Thermo Fisher Scientific; 00‐4958‐02).

### Imaging and quantification

Sections were imaged on a Zeiss Axio Scan.Z1 slide scanner (Carl Zeiss Microscopy, Inc., Dublin, CA, USA) at magnification ×20 using filters for DAPI, and green, red, and far‐red fluorescent proteins. The region of interest (ROI) was selected by measuring 5 mm from the tibial plateau and selecting a 2‐mm‐long region including the muscle, cortical bone, and some marrow (Fig. 1*B*). Images were exported as TIFF files and converted to Bioquant Imaging Files (BIFs) using the correct resolution (0.325 μm/pixel). BIOQUANT OSTEO software (Bioquant Image Analysis Corporation, Nashville, TN, USA) was used to measure the bone surface length, calcein length, and to count individual cells (blinded to sample identity and group). Cells within different regions of interest were counted separately (Fig. 1C). First, cells residing directly on the bone surface were counted as “bone surface” cells (Fig. 1C). Next, cells residing between the calcein label and bone surface were counted as “new bone” cells. Last cells within the periosteum between the first layer of bone surface cells and the muscle were counted as “periosteal” cells. Each cell was identified as either TdT^−^EdU^−^, TdT^+^EdU^−^, TdT^−^EdU^+^, or TdT^+^EdU^+^ to determine lineage specificity and whether the cell proliferated or was the product of proliferation, during the experiment.

### Dynamic histomorphometry

**Fig. 2:**
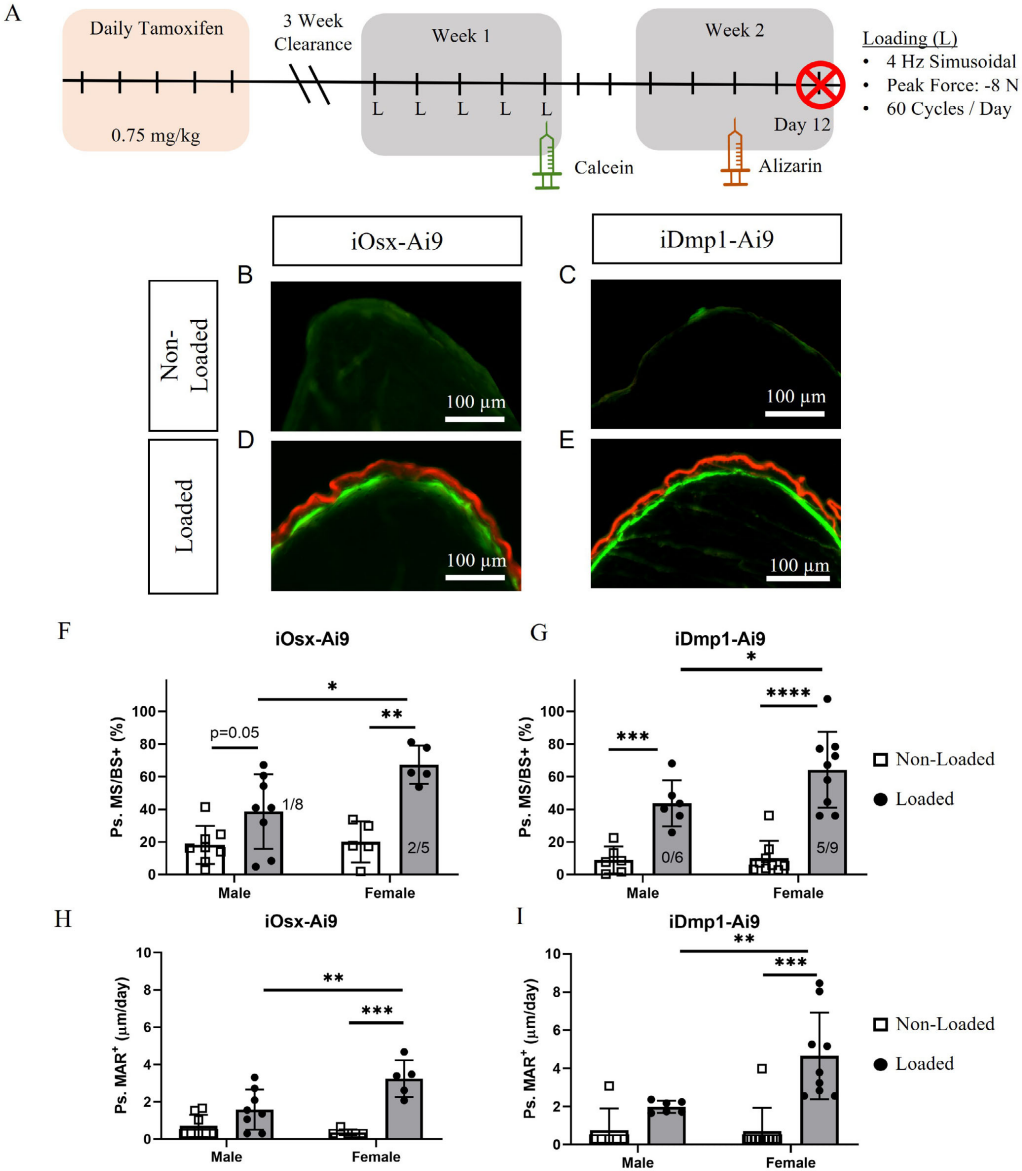
Dynamic histomorphometry of iOsx‐Ai9 and iDmp1‐Ai9 mice loaded to −8 N. (*A*) Timeline for dynamic histomorphometry protocol. (*B–E*) Representative images of (*B*,*C*) non‐loaded and (*D–E*) loaded iOsx‐Ai9 (males *n* = 8; females *n* = 5) and iDmp1‐Ai9 (males *n* = 6; females *n* = 9) limbs at the site of peak compressive strain on day 12 following the onset of loading. (*F*,*G*) Ps.MS/BS+ quantified for (*F*) iOsx‐Ai9 and (*G*) iDmp1‐Ai9. Fractions denote the incidence of woven bone. (*H*,*I*) Ps.MAR+ quantified for (*H*) iOsx‐Ai9 and (*I*) iDmp1‐Ai9. *p < 0.05, **p < 0.01, ***p < 0.001, ****p < 0.0001 by two‐way ANOVA repeated measures, Sidak multiple comparisons correction (factors: loading, sex). Ps.MAR+ = periosteal mineral apposition rate; Ps.MS/BS = periosteal mineralizing surface.

A subset of mice were loaded to a peak force of −8 N (iOsx‐Ai9: 8 males, 5 females; iDmp1‐Ai9: 6 males, 9 females) with the same loading protocol described above in order to confirm an anabolic response in these transgenic lines (Fig. 2*A*). These mice did not receive EdU. Calcein green was injected (ip) on day 5 of loading. Mice were allowed cage free activity for 1 week following the last loading bout. A second fluorochrome injection (alizarin complexone, 30 mg/kg ip; MilliporeSigma) was delivered to the mice on day 10 and mice were euthanized on day 12 from the start of loading.

Samples were kept in 70% ethanol until ethanol gradation steps, then transitioned to xylene and methyl methacrylate for plastic embedding. Samples were cut on a saw microtome (Leica Microsystems, Inc., Buffalo Grove, IL, USA) at the desired maximal compressive region (~5 mm proximal to the tibial‐fibular junction). Sections were cut transversely at approximately 100 μm thickness and polished with sandpaper to ~30 μm thickness. Sections were imaged on an Axio Imager (Carl Zeiss) on the 10× objective. Images were stitched together using Image Composite Editor (Microsoft Corp., Redmond, WA, USA). Bioquant OSTEO software was used to measure calcein and Alizarin labels in the samples according to standard histomorphometry guidelines.^(^ ^25^ ^)^ For samples with no apparent double label (iDmp1‐Ai9 non‐loaded: males *n* = 5, females *n* = 8; loaded: *n* = 0, iOsx‐Ai9 non‐loaded: males *n* = 5, females *n* = 4; loaded: males *n* = 2, females *n* = 0), 0.3 μm/day was used for mineral apposition rate (MAR) to compute bone formation rate (BFR).^(^ ^25^ ^)^ Woven bone mineralizing surface was added to bone mineralizing surface (MS/BS) measures to compute total MS/BS, and woven area thickness was used to modify MAR as described by Holguin and colleagues.^(^ ^23^ ^)^

### Statistics

The majority of outcomes were tested for significance by two‐way ANOVA with factors being loading and force (Prism; GraphPad Software, Inc., La Jolla, CA, USA). Contralateral non‐loaded limbs served as controls and were treated as repeated measures in the two‐way ANOVA. Post hoc Sidak tests with multiple comparison corrections were used to determine significance between groups. Lineage and proliferation outcomes from females and males were analyzed separately; sex was not tested for significance in lineage outcomes because of slight differences in strain stimulus, and in observed dynamic histomorphometry outcomes. Strain gauging data was analyzed by linear regression. For certain outcomes, one‐way ANOVA or *t* tests were performed as appropriate and are reported within figure captions. Statistical significance was defined as *p* < 0.05, with nonsignificant differences noted if *p* < 0.10. Bar graphs represent data as mean ± standard deviation (SD).

## Results

### Modest to robust levels of bone formation were induced by tibial loading in reporter mice

To assess bone formation in a subset of reporter mice, we used classical dynamic histomorphometry after loading to a peak force of −8 N. We observed increased bone formation on the periosteal surface of loaded tibias, with some woven bone, in iOsx‐Ai9 and iDmp1‐Ai9 mice (Fig. 2B‐I, Table S1). Robust bone formation was induced by −8 N and the incidence of woven bone formation (8/28) at this force indicated that −7 and −10 N were appropriate forces to generate mostly lamellar and woven bone formation responses, respectively, for subsequent lineage tracing experiments. Strain‐matched loading induced a greater anabolic response (periosteal MS/BS+ [Ps.MS/BS+] and MAR+) in female mice of both reporter lines. Thus, subsequent analyses were conducted separately for males and females.

**Fig. 3:**
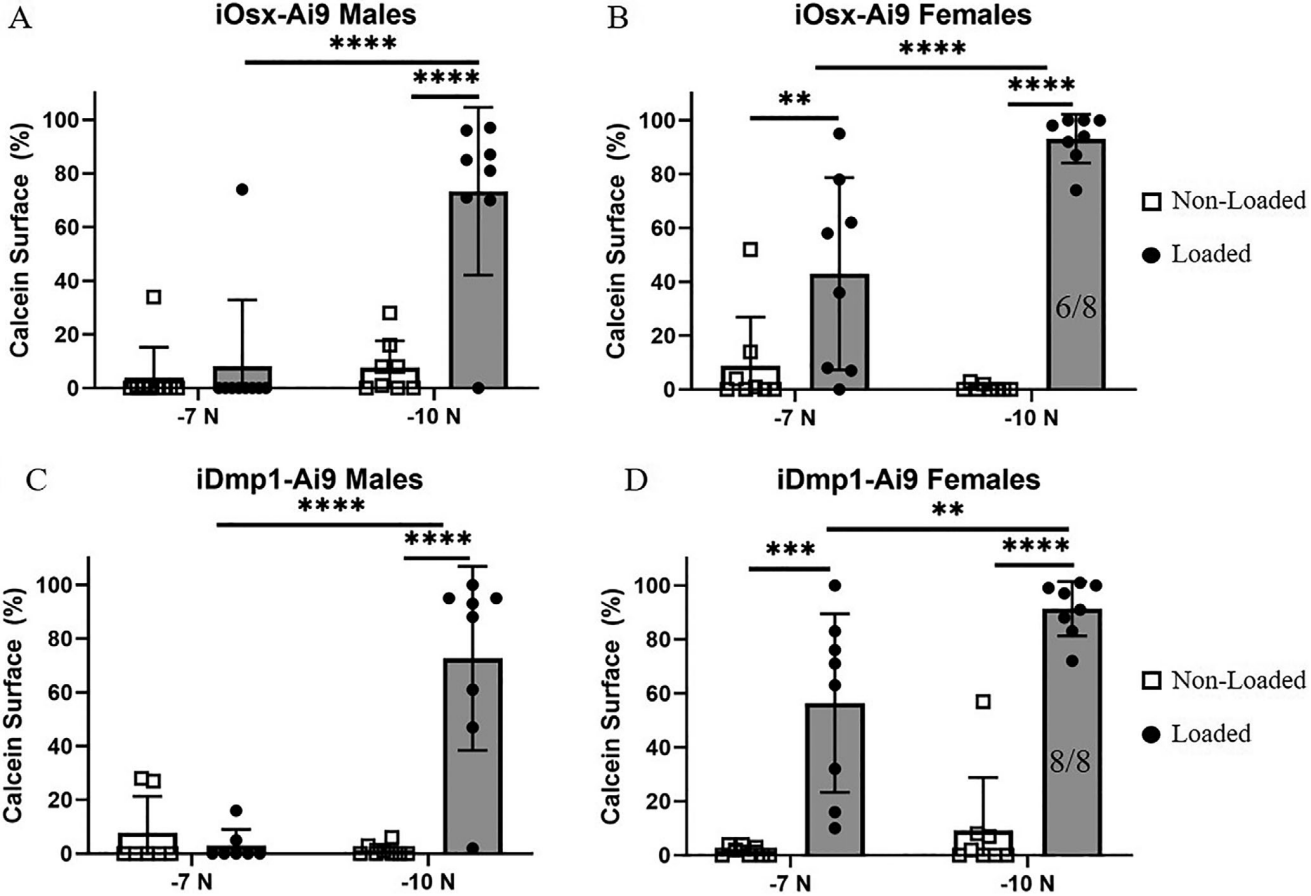
Calcein was incorporated at the periosteal surface of loaded bones from most groups, indicating increased mineralization in response to loading. Percentage of the bone surface that incorporated calcein into new bone in iOsx‐Ai9 (*A*) males (*n* = 8/force) and (*B*) females (*n* = 8/force) and in iDmp1‐Ai9 (*C*) males (−7 N, *n* = 7; −10 N, *n* = 8) and (*D*) females (*n* = 8/force). Fractions denote the incidence of woven bone. *p < 0.05, **p < 0.01, ***p < 0.001, ****p < 0.0001 by two‐way ANOVA repeated measures, Sidak multiple comparisons correction (factors: loading, force).

Although dynamic histomorphometry confirmed loading‐induced bone formation, that result is based on a protocol that applied −8 N peak force, with samples collected 12 days after the onset of loading, and analysis of double labels on 30‐μm transverse plastic sections (Fig. 2*A*). We next analyzed bone formation from samples used for lineage tracing, which were loaded to −7 or −10 N, collected on day 8, and analyzed based on a single calcein label on 6‐μm longitudinal cryosections (Fig. 1*A*). In female mice, we observed a significant increase in calcein‐labeled surface in both iOsx‐Ai9 and iDmp1‐Ai9 limbs loaded to −7 and −10 N (Fig. 3B,D). Greater calcein label was apparent in female mice loaded to −10 N (iOsx‐Ai9 93%, iDmp1‐Ai9 91%) compared to −7 N (iOsx‐Ai9 43%, iDmp1‐Ai9 56%), consistent with a greater strain stimulus. In contrast, in male mice, only −10‐N loading increased calcein surface at this time point in the ROI (Fig. 3A,C). Calcein labeled 73% of the bone surface in male iOsx‐Ai9 and iDmp1‐Ai9 mice loaded to −10 N. Notably, woven bone formation was apparent at this time point in six of eight iOsx and eight of eight iDmp1 female mice loaded to −10 N, whereas no instances of woven bone were observed in male mice.

In addition to analysis of fluorochrome labels, we also quantified cell number at the bone surface and within the periosteum (cells not directly on the bone surface) to detect whether loading induced expansion of these cell populations. Only female mice loaded to −10 N experienced an increase in cell number at the bone surface (iOsx‐Ai9 14%, iDmp1‐Ai9 19%) (Fig. S2). This effect was even greater in the periosteum, where female mice loaded to −10 N experienced a 160% increase in cell number in iOsx‐Ai9 mice and a 220% increase in iDmp1‐Ai9 mice. Taken together, these data demonstrate that our protocol induces modest (−7 N) and robust (−10 N) levels of bone formation in these mice using standard dynamic histomorphometry, and samples directly used for subsequent lineage tracing.

### TdTomato was leaky in 11% of surface cells of iDmp1‐Ai9 mice

Bones of iOsx‐Ai9 and iDmp1‐Ai9 mice should not show TdTomato expression unless dosed with tamoxifen. As expected, in iOsx‐Ai9 mice without TAM, TdT^+^ cells were rarely found on the bone surface in non‐loaded (0.3%) or loaded limbs (0.5%) (Fig. S3). However, in iDmp1‐Ai9 mice without TAM, TdT^+^ cells were found in 11% of bone surface cells in non‐loaded limbs, and 2% of cells in loaded limbs. No TdT^+^ cells were identified in Cre^−^ mice. Thus, there is modest leakiness on periosteal surface in iDmp1‐Ai9 mice that is not attributed to loading. Notably, we observed approximately half of the osteocytes in iDmp1‐Ai9 mice to be TdT^+^ without TAM administration; we quantified this on a small set of samples (non‐loaded, *n* = 2; loaded, *n* = 2) where expression was identified in 43% to 70% of osteocytes with no clear loading effect. We did not detect any off‐target expression of TdTomato in the marrow or muscle cells in iOsx‐Ai9 or iDmp1‐Ai9 mice with or without tamoxifen administration.

### Preexisting Osx‐lineage cells are a primary source of cells for −7 N loading stimulus but play less of a role in maintaining bone formation for −10 N stimulus

**Fig. 4:**
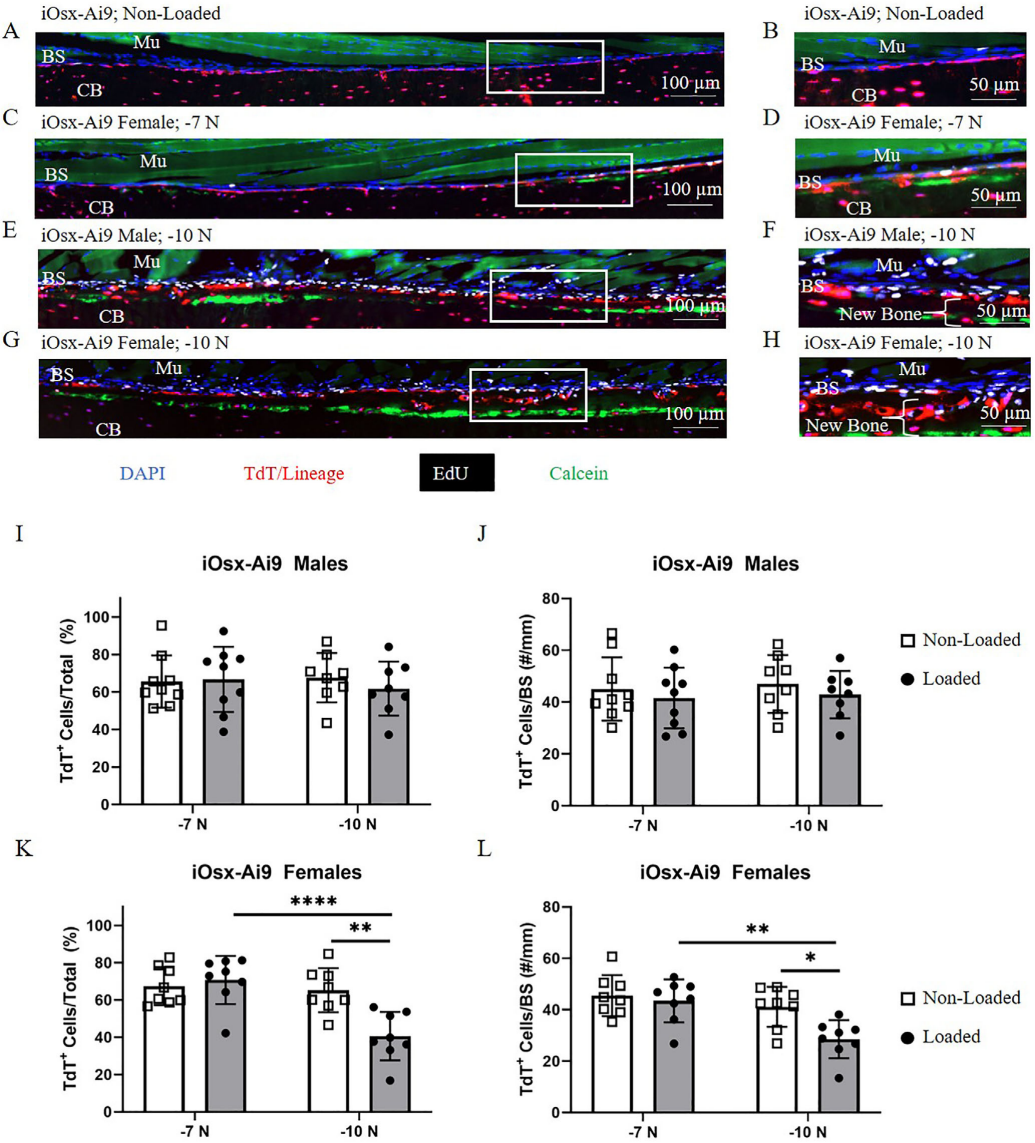
Contribution of iOsx‐Ai9 TdT^+^ cells to bone formation. (*A–H*) Representative images of iOsx‐Ai9 (*A*,*B*) non‐loaded limbs, (*C*,*D*) female limbs loaded to −7 N, (*E*,*F*) male limbs loaded to −10 N, and (*G*,*H*) female limbs loaded to −10 N. Male limbs loaded to −7 N (not shown) were similar to non‐loaded. (*I–L*) Quantification of TdT^+^ cells at the bone surface in iOsx‐Ai9 (*I*,*J*) males and (*K*,*L*) females. *p < 0.05, **p < 0.01, ***p < 0.001, ****p < 0.0001 by two‐way ANOVA repeated measures, Sidak multiple comparisons correction (factors: loading, force). BS = bone surface; Mu = muscle; CB = cortical bone.

The percentage and number of TdT^+^ cells at the bone surface in male and female iOsx‐Ai9 mice did not change with loading to −7 N, but was diminished in female mice loaded to −10 N (Fig. 4A‐L). Approximately 65% of cells on the non‐loaded bone surface of males and females were TdT^+^. These cells persisted on the surface with loading in male mice, regardless of force. A similar persistence of TdT^+^ cells on the bone surface occurred in female mice loaded to −7 N. In contrast, in female mice loaded to −10 N there were 29% fewer TdT^+^ cells on the bone surface of loaded versus non‐loaded tibias (41 versus 65%; 29 versus 41 cells/mm). Thus, after a higher load stimulus (−10 N) that induced woven bone in female mice, there is a depletion of pre‐existing Osx‐lineage cells on the bone surface, suggesting replacement by newly differentiated osteoblasts. Quantification of TdT^+^ cells on calcein^+^ surfaces (or calcein^‐^ surfaces) showed similar results as described here for the whole bone surface within the region of interest (Fig. S4, Table S2). We also calculated the number of TdT^+^ cells on calcein^+^ surfaces as percent of total TdT^+^ cells on the bone surface (Fig. S5). Overall, there was no clear evidence that TdT^+^ cells were either enriched or depleted at sites of calcein label.

### Preexisting Dmp1‐lineage cells are a primary source of cells for −7 N loading stimulus but are depleted markedly on day 8 following −10 N loading

**Fig. 5:**
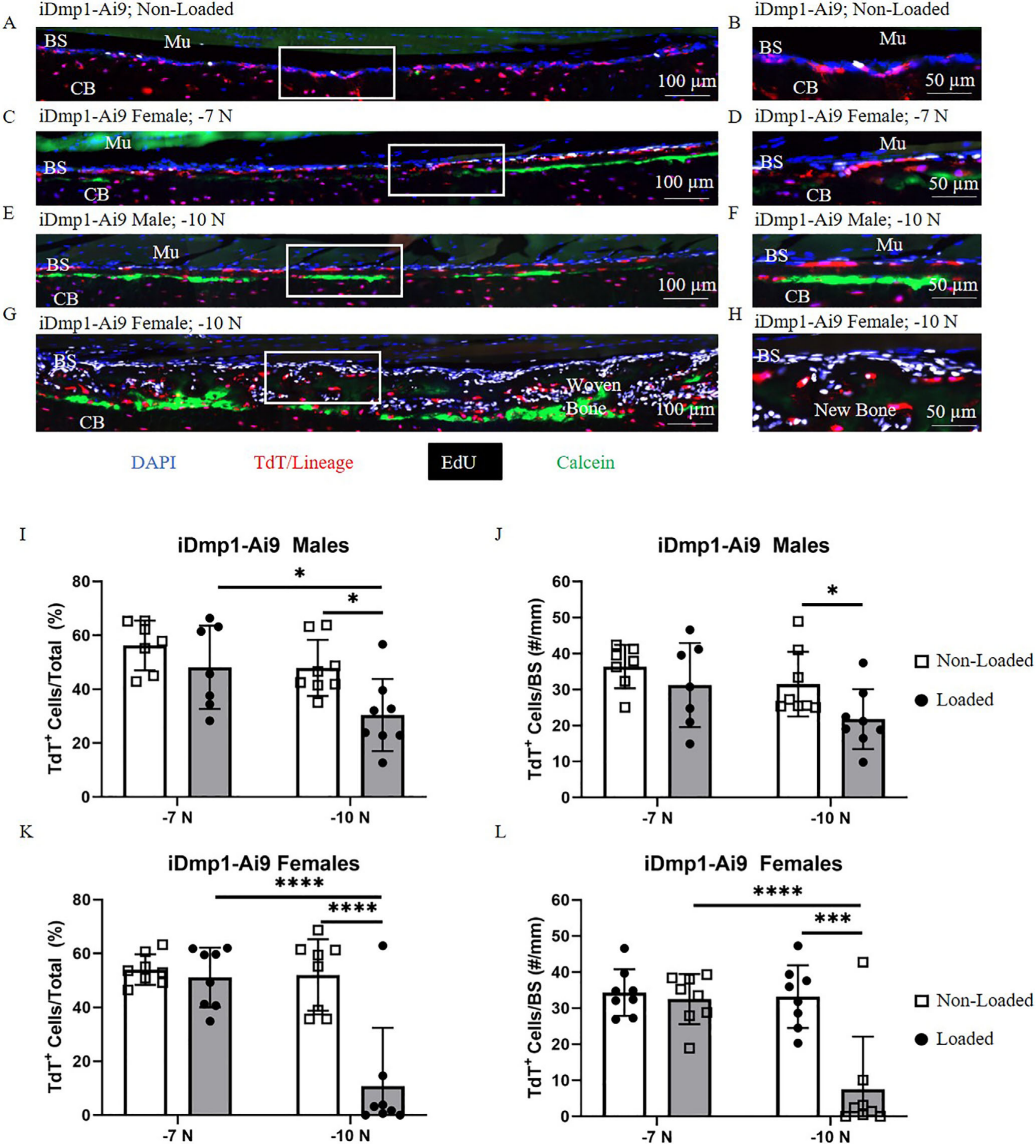
Contribution of iDmp1‐Ai9 TdT^+^ cells to bone formation. (*A–H*) Representative images of iDmp1‐Ai9 (*A*,*B*) non‐loaded limbs, (*C*,*D*) female limbs loaded to −7 N, (*E*,*F*) male limbs loaded to −10 N, and (*G*,*H*) female limbs loaded to −10 N. Male limbs loaded to −7 N (not shown) were similar to non‐loaded. (*I–L*) Quantification of TdT^+^ cells at the bone surface in iDmp1‐Ai9 (*I*,*J*) males and (*K*,*L*) females. *p < 0.05, **p < 0.01, ***p < 0.001, ****p < 0.0001 by two‐way ANOVA repeated measures, Sidak multiple comparisons correction (factors: loading, force). BS = bone surface; Mu = muscle; CB = cortical bone.

Similar to iOsx‐Ai9, the percentage and number of TdT^+^ cells at the bone surface of iDmp1‐Ai9 mice were similar in non‐loaded and loaded limbs at −7 N, but these cells decreased by day 8 in limbs loaded to −10 N (Fig. 5A‐L). In non‐loaded limbs, TdT^+^ cells made up approximately 50% of cells at the bone surface. No change in percentage or number of TdT^+^ cells at the surface occurred in either male or female mice loaded to −7 N. In contrast, in males loaded to −10 N there were 31% fewer TdT^+^ cells on the surface of loaded versus non‐loaded tibias (30% vs. 48%; 22 vs. 32 cells/mm). A more pronounced effect was found in female mice loaded to −10 N, resulting in 78% fewer TdT^+^ cells at the bone surface of loaded versus non‐loaded tibias (11% vs. 52%, 8 vs. 33 cells/mm). Thus, after a potent loading stimulus (−10 N) that induces robust bone formation, there is a depletion of preexisting Dmp1‐lineage cells on the bone surface that is more pronounced than the depletion of Osx‐lineage cells.

### Proliferation contributes more to the bone surface cell population at −10‐N loading stimulus

**Fig. 6:**
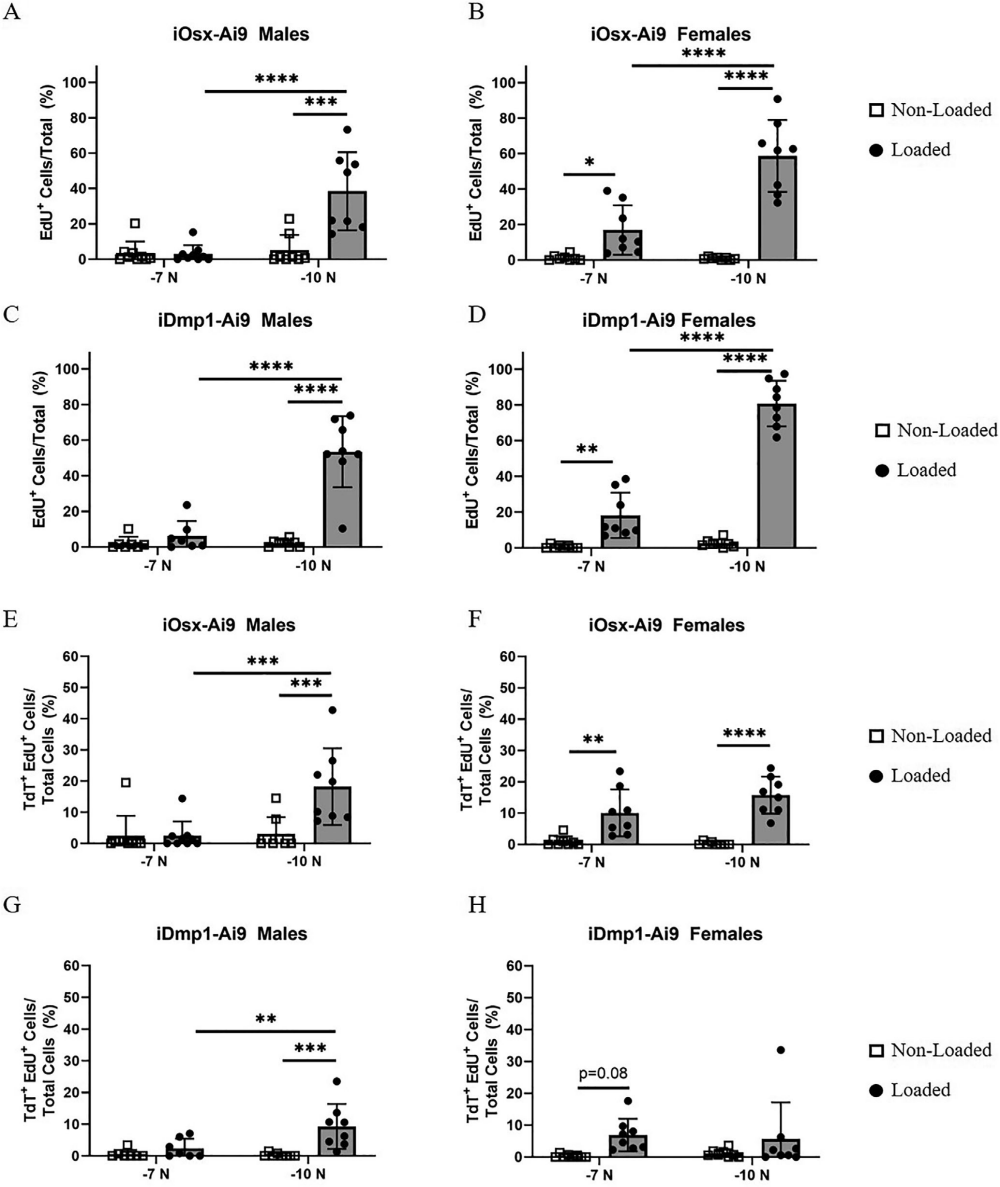
Contribution of proliferation to bone surface cell population. (*A*–*D*) Total percentage of bone surface cells that proliferated or arose via proliferation (EdU^+^) in iOsx‐Ai9 (*A*) males and (*B*) females, and iDmp1‐Ai9 (*C*) males and (*D*) females. (*E*–*H*) Percentage of cells on the bone surface that proliferated or arose via proliferation from a lineage positive cell (TdT^+^EdU^+^) in iOsx‐Ai9 (*E*) males and (*F*) females, and iDmp1‐Ai9 (*G*) males and (*H*) females. *p < 0.05, **p < 0.01, ***p < 0.001, ****p < 0.0001 by two‐way ANOVA repeated measures, Sidak multiple comparisons correction (factors: loading, force).

At the bone surface, cells that proliferated or arose via proliferation (EdU^+^) were rare in non‐loaded bones. Loading induced cell proliferation in male mice loaded to −10 N and female mice loaded to either force (Fig. 6*A*–*D*). Of these groups, female mice loaded to −7 N had a modest percentage of EdU^+^ cells on the bone surface (17% iOsx‐Ai9, 18% iDmp1‐Ai9). Proliferation contributed more at the higher (−10 N) force level in both male mice (38% iOsx‐Ai9, 53% iDmp1‐Ai9) and female mice (59% iOsx‐Ai9, 81% iDmp1‐Ai9). Similar trends were observed in number of EdU^+^ cells per bone surface, with the maximum number of EdU^+^ cells occurring in iDmp1‐Ai9 female mice loaded to −10 N (62 cells/mm) (Fig. S5A‐D).

Some of the cells that proliferated in response to loading were also lineage‐positive (Fig. 6*E–H*). On tibias of iOsx‐Ai9 female mice loaded to −7 N, 10% of bone surface cells were TdT^+^EdU^+^. On tibias of iOsx‐Ai9 mice loaded to −10 N, the TdT^+^EdU^+^ cell fraction was 16% in females and 18% in males. By comparison, there were fewer TdT^+^EdU^+^ cells on the bone surface of iDmp1‐Ai9 mice. In limbs of iDmp1‐Ai9 mice loaded to −7 N, the percentage of TdT^+^EdU^+^ cells was <5% and not different from non‐loaded limbs, whereas in limbs loaded to −10 N the percentage was <10%. Similar effects occurred in number of TdT^+^EdU^+^ cells normalized to bone surface length, with the maximum number of cells occurring in iOsx‐Ai9 male mice loaded to −10 N (13 cells/mm) (Fig. S5E–H).

Although TdT^+^EdU^+^ cells comprised <20% of all bone surface cells on loaded limbs, the contribution of proliferation to the lineage positive cell pool was significant. Of the cells that were lineage positive on the bone surface, the percentage that proliferated or arose via proliferation was greatest in female mice loaded to −10 N (41% iOsx‐Ai9, 46% iDmp1) (Fig. S5). However, as noted above (Figs. 4 and 5) the total number of TdT^+^ cells in these loaded limbs was low (29 TdT^+^ cells/mm iOsx‐Ai9, 8 TdT^+^ cells/mm iDmp1‐Ai9). In male mice loaded to −10 N, 30% of TdT^+^ cells arose via proliferation at the bone surface (43 TdT^+^ cells/mm iOsx‐Ai9, 22 TdT^+^ cells/mm iDmp1‐Ai9). Finally, a small portion of TdT^+^ cells in iOsx‐Ai9 female mice loaded to −7 N arose via proliferation (15%, 43 TdT^+^ cells/mm). Taken together, the results show that proliferation at the bone surface increased with increasing applied force (strain), and that many lineage‐positive cells (15%–46%) proliferated or arose via proliferation.

### In tibias loaded to −10 N, the majority of newly embedded cells are TdT ^+^

**Fig. 7:**
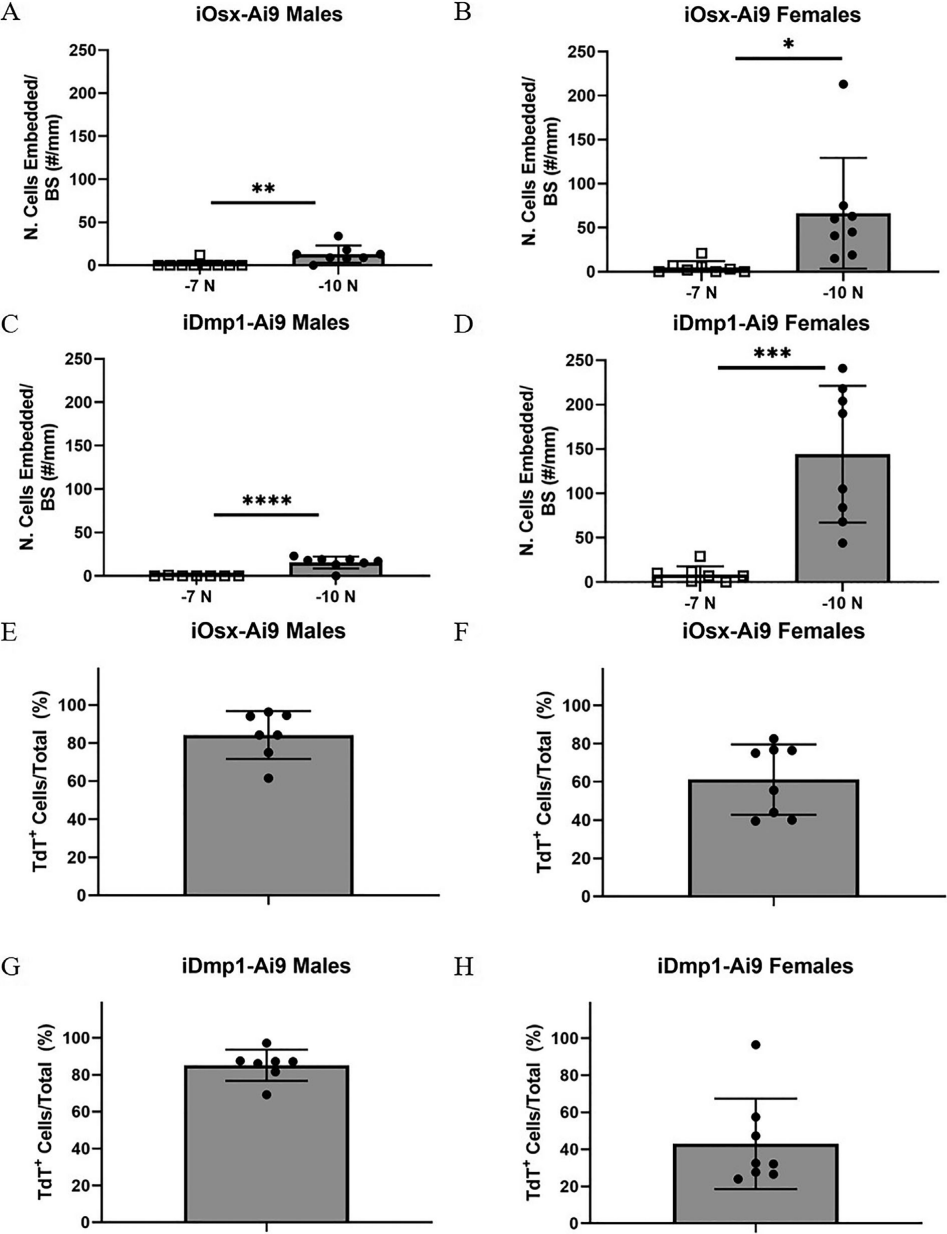
Cell population within newly formed bone. (*A–D*) Number of cells that were incorporated into new bone, normalized to bone surface length in iOsx‐Ai9 (*A*) males and (*B*) females, and iDmp1‐Ai9 (*C*) males and (*D*) females. (*E*–*H*) Percentage of embedded cells that are TdT^+^ in iOsx‐Ai9 (*E*) males and (*F*) females, and iDmp1‐Ai9 (*G*) males and (*H*) females. *p < 0.05, **p < 0.01, ***p < 0.001, ****p < 0.0001 by unpaired *t* test.

Cells located between the calcein label (day 5) and the bone surface (day 8) were interpreted as newly embedded osteoblasts/osteocytes. A negligible number of newly embedded cells were observed in mice loaded to −7 N, whereas a few cells (13–16 cells/mm) were embedded in this region in male mice loaded to −10 N (Fig. 7A‐D). Notably, in the male mice loaded to −10 N, the majority of embedded cells were TdT^+^ (84% iOsx‐Ai9; 85% iDmp1‐Ai9; Figs. 7E,G); these proportions were comparatively higher than the percentage of lineage‐positive cells on the bone surface of non‐loaded limbs (68% iOsx‐Ai9; 48% iDmp1‐Ai9; Figs. 4*I* and 5*I*). Female mice loaded to −10 N, which had the highest proportion of woven bone, had by far the greatest number of cells embedded (66 cells/mm iOsx‐Ai9; 144 cells/mm iDmp1‐Ai9). These mice also had a high proportion of lineage‐positive embedded cells (61% iOsx‐Ai9, 43% iDmp1‐Ai9; Figs. 7F,H); these proportions are comparable to the percentage of lineage‐positive cells in non‐loaded limbs (65% iOsx‐Ai9; 52% iDmp1‐Ai9; Figs. 4*K* and 5*K*). Thus, lineage‐positive cells are the main source of newly embedded cells, especially for lamellar bone formation.

### The periosteum is composed primarily of TdT ^−^ cells that are more proliferative at −10‐N loading stimulus

**Fig. 8:**
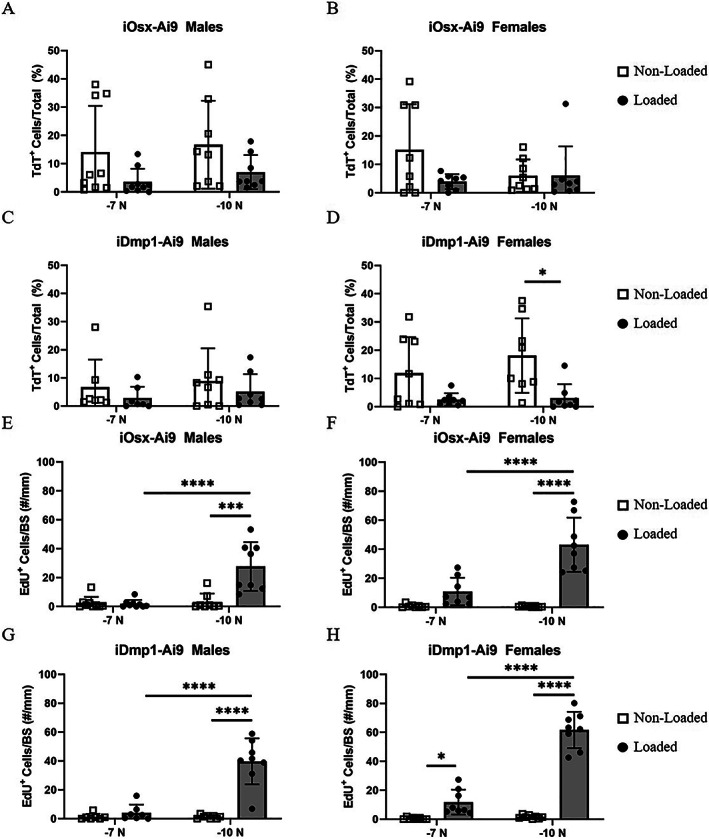
Periosteum lineage positive population and proliferative response with loading. (*A–D*) Percentage of periosteal cells that are TdT^+^ in iOsx‐Ai9 (*A*) males and (*B*) females, and iDmp1‐Ai9 (*C*) males and (*D*) females. (*E–H*) Percentage of periosteal cells that are EdU^+^ in iOsx‐Ai9 (*E*) males and (*F*) females, and iDmp1‐Ai9 (*G*) males and (*H*) females. *p < 0.05, **p < 0.01, ***p < 0.001, ****p < 0.0001 by two‐way ANOVA repeated measures, Sidak multiple comparisons correction (factors: loading, force).

Within the periosteum (cells not directly on the bone surface), the majority of cells were lineage negative (Fig. 8*A*–*D*). TdT^+^ cells comprised only 6% to 17% of the periosteum in non‐loaded limbs of iOsx‐Ai9, and 7% to 18% in iDmp1‐Ai9 mice. Loading caused a decrease in percentage of TdT^+^ cells in the periosteum of female iDmp1‐Ai9 mice loaded to −10 N (non‐loaded 18%, loaded 3%). However, this group experienced the greatest increase in total number of cells/mm in the periosteum (216% increase).

There were few proliferative cells in the periosteum of non‐loaded limbs (average EdU^+^/Total <5%; Fig. 8*E*–*H*). Loading stimulated increases in periosteal proliferation only in mice loaded to −10 N. The percentage of cells that were EdU^+^ in male mice loaded to −10 N increased to 33% in iOsx‐Ai9 mice and 45% in iDmp1‐Ai9 mice. An even greater portion of cells were EdU^+^ in female mice loaded to −10 N (iOsx‐Ai9 56%, iDmp1‐Ai9 58%). Thus, the majority of periosteal cells are not committed to the Osx or Dmp1 lineage, and a proportion are induced to proliferate upon loading, especially at higher forces.

## Discussion

Few studies have investigated the differentiation stage of cells contributing to bone formation induced by mechanical loading, despite many advances in lineage tracing tools, imaging techniques, and loading models for in vivo use. Recent studies have identified roles for osteoprogenitors (Prx1^+^),^(^ ^9^, ^10^ ^)^ but whether these cells contribute directly to the bone forming surface, and whether they are important for initiating bone formation versus sustaining the response is unclear. Others have shown that more differentiated cells (Osx‐lineage) contribute to the initial bone formation response (day 5 of lamellar loading),^(^ ^7^ ^)^ but this reporter mouse marks cells in several stages of osteoblast differentiation. We sought to clarify the role of preexisting Osx‐expressing cells in sustaining the bone formation response at day 8 of lamellar loading. We also investigated the role of mature osteoblasts, using the iDmp1‐Ai9 mouse, to better delineate the population of cells contributing to bone formation. We find that preexisting Osx and Dmp1 lineage cells are the main contributors to modest lamellar bone formation, but their contribution diminishes for more moderate lamellar and robust woven bone formation.

We designed our study to extend the prior work on lineage tracing after bone loading. We loaded male and female mice to two forces (−7 N, −10 N) to stimulate a range of anabolic responses. These forces engendered peak compressive strains of approximately −2200 and −3200 με, respectively, and previous work in 5‐month‐old female C57Bl/6 mice has shown that this range of applied strain levels induces a range of responses from lamellar to woven.^(^ ^23^ ^)^ In the current study this resulted in three degrees of bone formation: modest lamellar, moderate lamellar, and robust woven. The degrees of bone formation in iOsx‐Ai9 and iDmp1‐Ai9 reporter mice were similar. Labeling of calcein at the initiation of bone formation was found to be helpful for interpretation of the type of bone formation present in each sample. Finally, continuous EdU administration during this time period revealed a dose‐response in terms of increasing proliferation with increasing degrees of bone formation. Notably, we also found small contributions of Osx and Dmp1 lineage cell proliferation to the bone forming surface, although their contribution did not increase with more bone formation and remained below 20% of total cells at the bone surface.

**Fig. 9:**
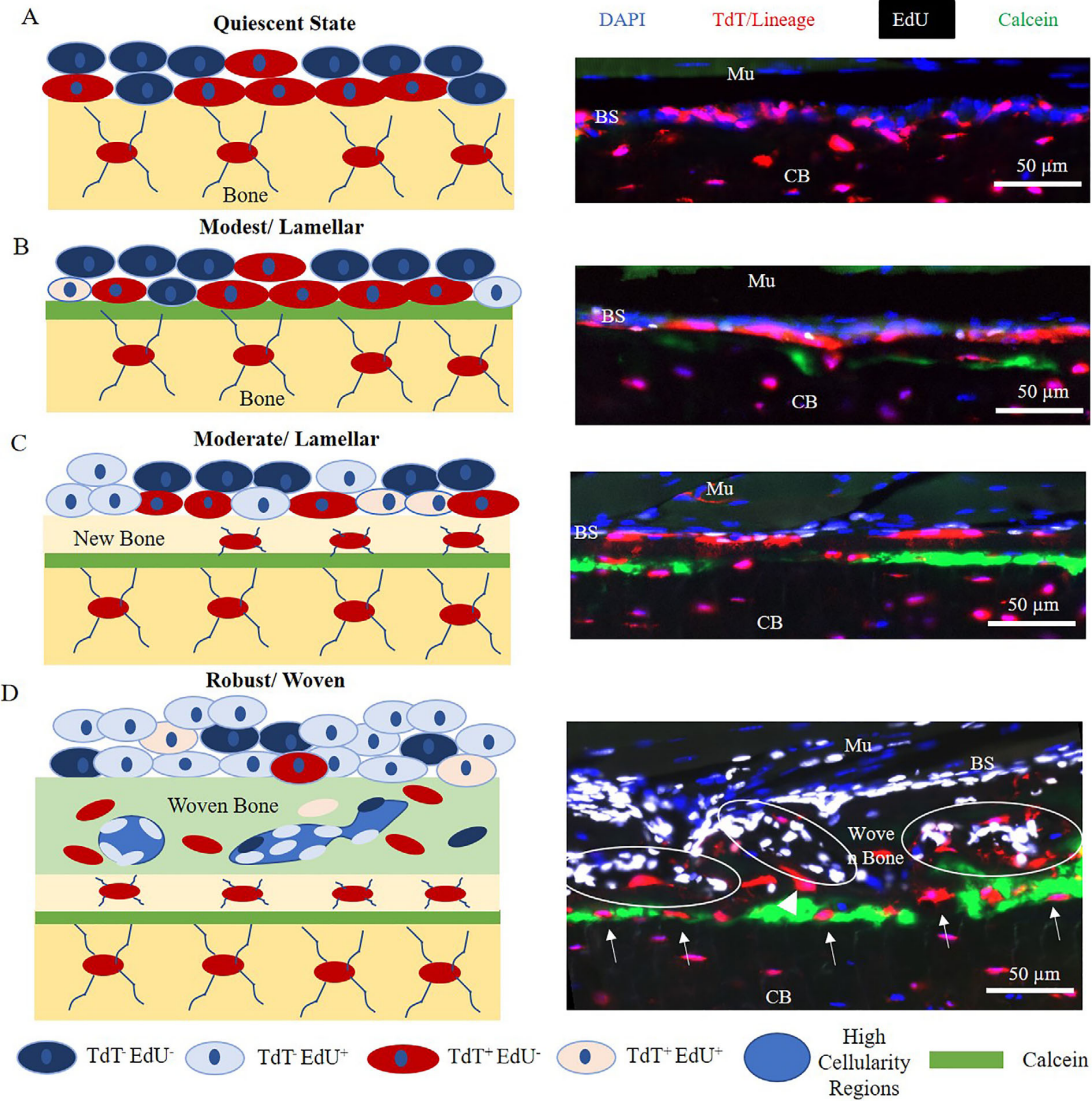
Model depicting bone formation responses to loading. (*A*) A major component of the quiescent bone surface includes Osx and Dmp1 lineage cells (shown as TdT^+^/red). (*B*) Modest stimulation induces lamellar bone formation characterized by a thin calcein layer near the bone surface, with Osx and Dmp1 lineage cells contributing to the majority of the response. Some proliferation occurs at the bone surface. (*C*) Moderate stimulation induces lamellar bone formation with a thicker area of new bone between the calcein label and bone surface. Proliferation of less differentiated cells contributes to this response (TdT^−^EdU^+^). Dmp1 lineage cells decrease at the bone surface, while these cells can be found populating the new bone region. (*D*) Robust stimulation induces woven bone formation characterized by circular regions of high cellularity (outlined in white), an expansion of the periosteum, increased cell number at the bone surface, and many cells in the new bone area. A decrease in Osx lineage cells occurs and Dmp1 lineage cells are nearly depleted from the bone surface.

The types of bone formation that occurred in the groups analyzed in this study were of a modest lamellar type (females loaded to −7 N), a moderate lamellar type (males loaded to −10 N), and a robust woven type (females loaded −10 N) (Fig. 9A‐D). In the first scenario (Fig. 9B), cells can be found residing directly atop the calcein surface, similar to results found by Zannit and Silva^(^ ^7^ ^)^ where preexisting Osx‐lineage cells lined nearly all of the bone surface in loaded and non‐loaded limbs. The modest loading stimulation appears to activate cells already at the bone surface, which are mostly Osx^+^ and Dmp1^+^ cells. No increases in cell number, and a modest increase in proliferation (17%–18%) were found at this stage. In the second scenario (Fig. 9C), a balanced exchange of cells takes place between the periosteum, bone surface, and new bone regions. Dmp1^+^ cells appear to be poised to embed into newly formed bone between calcein and the bone surface, causing a decrease in TdT^+^ cells at the bone surface. However, no overall change in cell number occurs. Thus, cells that embed are replaced by TdT^−^ cells via proliferation (38%–53% EdU^+^) and/or recruitment from the periosteum. In the last scenario (Fig. 9D), a highly cellular region appears below the bone surface. This region has many cell types, whereas few Dmp1^+^ cells remain at the bone surface (<10%) following this type of bone formation. Unlike the other cases, an increase in cell number at the surface and in the periosteum occurs, composed primarily of TdT^−^ cells (>90%). The new bone region has woven bone characteristics with regions densely occupied by TdT^−^ cells, consistent with bone marrow. Overall, it appears the depletion of preexisting Osx and Dmp1 lineage cells, especially Dmp1 lineage cells, occurs and that these cells are replaced at the surface by presumably less‐differentiated cells. These cells could be of Prx1 lineage, supporting work by Moore and colleagues^(^ ^10^ ^)^ and Cabahug‐Zuckerman and colleagues.^(^ ^9^ ^)^ Although our results show that both Osx and Dmp1 lineage cells contribute to the bone formation response, their relative degrees of contribution cannot be quantitatively compared due to differences in the labeling efficiencies of the two reporter lines, and modest differences in mechanical strains and responsiveness to loading.

Although iOsx‐Ai9 and iDmp1‐Ai9 mice reportedly mark cells beginning at earlier and later stages of osteoblast differentiation,^(^ ^12^, ^13^, ^14^, ^17^, ^26^ ^)^ respectively, we found many similarities between the labeling of cells in these models. Specifically, iOsx‐Ai9 only labeled ~10% more cells compared to iDmp1‐Ai9 in non‐loaded tibias. This suggests that the majority of cells (at least 48%–56%) on the quiescent bone surface could be mature osteoblasts. However, many differences between the mouse models exist, including leakiness, efficiency in labeling, tissue level strains, and responses to loading. Others have also reported labeling on the bone surface in iDmp1‐Ai9 mice, but not to this extent.^(^ ^12^, ^13^, ^14^ ^)^ Differences in timing and dosages of tamoxifen, clearance periods, and ages of mice are known to effect degree of TdT expression.^(^ ^12^, ^13^ ^)^ The percentage of cells marked by Osx‐lineage on day 8 in this study were lower than in non‐loaded tibias on day 5 of loading found in Zannit and Silva.^(^ ^7^ ^)^ However, the iOsx‐Ai9 mouse line used here was less leaky, with fewer than 1% labeled on the bone surface whereas Zannit and Silva^(^ ^7^ ^)^ reported 20% of cells on the surface labeled without tamoxifen administration. We note that the iOsx‐Ai9 mouse line used here was started by mating new OsxCreERT2 and Ai9 breeders, and thus was different from the line used by Zannit and Silva.^(^ ^7^ ^)^ Finally, we note that the iDmp1‐Ai9 mouse line used here showed some leakiness, with 11% of periosteal cells and ~50% of osteocytes labeled (TdT^+^) without tamoxifen (Fig. S3B,F).

Calcein labeling at the initiation of bone formation (days 4 and 5 of loading) was important in identifying degree and type of bone formation in this study. Although the force‐strain relationships for males and females were similar, we found varied responses noticeable by extent of bone formed between the calcein label and new bone surface. This qualitative difference was also exemplified by the percentage of surface labeled with calcein in the region of interest; 0% for males loaded to −7 N, 43% to 56% for females loaded to −7 N, 73% for males loaded to −10 N, and 91% to 93% for females loaded to −10 N. These sex differences were statistically significant by standard dynamic histomorphometry at day 12 following the start of loading. Although our strain gauging data demonstrated similar strains in male and female mice, others have shown that female C57Bl/6 mice experience more strain per a given force compared to males.^(^ ^27^ ^)^ Few studies have investigated differences between male and female mice, although Meakin and colleagues^(^ ^28^ ^)^ did show differences in bone response to loading between female and male mice with age. Identification of type of bone formation within the samples analyzed at this particular time point was important for interpretation of our results. For instance, at this time point six of eight and eight of eight females loaded to −10 N formed woven bone, whereas males loaded to the same force (and strain stimulus) were not actively forming woven bone. Results are presented here as differences in degrees of bone formation occurring, but these differences might be in part driven by differences in how males and females respond to the same strain stimulus.

Our study has some limitations. First, calcein labeling on frozen sections was not 100% efficient. Calcein may have been lost by processing in aqueous solutions, and signal intensity is less in thin (6 μm) sections compared to thicker plastic sections (30 μm). Thus, we observed some calcein‐negative surfaces directly adjacent to calcein‐positive surfaces with otherwise similar morphology and TdT labeling (e.g., Fig. 5C,E); some of these calcein‐negative surfaces were most likely active bone forming surfaces. This is consistent with the similar percent of TdT^+^ cells whether or not overlying a calcein label (Fig. S4, Table S2). An additional limitation to this study was not quantifying cell death (apoptosis), which represents another option for the cell fate of osteoblasts following bone formation. Future studies could perform experiments at multiple time points to better understand the role of cell death in osteoblast fate following loading‐induced bone formation.

In summary, our results show predominant contributions by preexisting Osx and Dmp1 lineage cells to loading‐induced bone formation, especially for lamellar bone formation. However, an increase of lineage‐negative cells also suggests that recruitment of other cells may be necessary for sustaining the bone formation response at day 8, especially for more robust types of bone formation, such as woven bone. Future studies are needed to determine if recruitment of earlier cell types contribute to sustained bone formation in the period after initial bone formation. It remains to be shown if lineage‐positive cells become completely depleted from the bone surface over longer periods. Importantly, we found differences in responses in male and female mice despite strain‐matched stimuli, although this allowed an analysis of varied bone formation responses.

## Conflict of Interests

MJS: Editorial Board *CTI*, *Bone*, *JOR*; Board of directors ORS. TLH: None. The authors declare there are no conflicts of interest.

### Peer Review

The peer review history for this article is available at https://publons.com/publon/10.1002/jbm4.10593.

## Supporting information


**Figure S1** Similar strains were engendered by 7 N and 10 N peak compressive forces across mouse strain and sex. Force‐strain relationship for (A) iOsx‐Ai9 (males *n* = 7; females *n* = 8) and (B) iDmp1‐Ai9 with respective linear regression lines are shown (males *n* = 5; females *n* = 7). (C) Linear regression equations for each group and the strains produced by 7 N and 10 N compressive forces. Higher strains (+180–260 με; +13%) were induced by these forces in female iOsx‐Ai9 mice compared to males; whereas the slope of the corresponding linear regression lines were not different, the y‐intercepts were significantly different (p = 0.0099).

**Figure S2** Loading induced an increase in cell number at the bone surface and periosteum. (A‐D) Quantification of total number of cells at the bone surface normalized to bone surface length in iOsx‐Ai9 (A) males and (B) females and iDmp1‐Ai9 (C) males and (D) females. (E‐H) Quantification of total number of cells within the periosteum (not directly adjacent to the bone surface) normalized to bone surface length in iOsx‐Ai9 (E) males and (F) females and iDmp1‐Ai9 (G) males and (H) females. *p < 0.05, **p < 0.01, ***p < 0.001, ****p < 0.0001 by two‐way ANOVA repeated measures, Sidak multiple comparisons correction (factors: loading, force).

**Figure S3** TdTomato leakiness in iOsx‐Ai9 and iDmp1‐Ai9 mice not treated with tamoxifen. (A,B) Representative images of leakiness of (A) iOsx‐Ai9 and (B) iDmp1‐Ai9 mice without tamoxifen administration in non‐loaded limbs. (C) Representative image of a Cre^−^ mouse where no TdT^+^ cells were found on the bone surface. (D,E) Quantification of the number of TdT^+^ cells on the bone surface normalized to total number of bone surface cells in (D) iOsx‐Ai9 and (E) iDmp1‐Ai9 mice that did not receive tamoxifen. (F) Percent TdT^+^ cells within the cortical bone of iDmp1‐Ai9 mice. **p < 0.01 by paired *t* test. Male and female data combined. No tamoxifen: iOsx‐Ai9 total *n* = 6 (males *n* = 3, females *n* = 3); iDmp1‐Ai9 total *n* = 8 (males *n* = 4, females *n* = 4). Cre Negative: iOsx‐Ai9 total *n* = 6 (males *n* = 3, females *n* = 3); iDmp1‐Ai9 total *n* = 7 (males *n* = 4, females *n* = 3).

**Figure S4** Contribution of TdT^+^ cells to bone formation was similar at sites with or without calcein label. Percent values are computed from ratios of number of cells with TdT expression adjacent to (i.e., overlying) calcein label (TdT^+^Calcein^+^ Cells) per total number of cells adjacent to calcein label (Calcein^+^ Cells), or TdT^+^ cells not adjacent to calcein label (TdT^+^Calcein^‐^ Cells), per total cells not adjacent to calcein label (Calcein^‐^Cells). (A‐D) Quantification of TdT^+^ cells adjacent to calcein^+^ surfaces in loaded limbs of iOsx‐Ai9 (A) males and (B) females, and iDmp1 (C) males and (D) females.(E‐H) Quantification of TdT^+^ cells adjacent to calcein^‐^ surfaces in loaded limbs of iOsx‐Ai9 (E) males and (F) females, and iDmp1 (G) males and (H) females. *p < 0.05, **p < 0.01, ***p < 0.001, ****p < 0.0001 by unpaired *t* test. Calcein^+^ Surfaces: One sample was removed from iOsx‐Ai9 –10 N male group and one sample removed from iOsx‐Ai9 –7 N female group due to no presence of calcein. Male −7 N groups not included due to minimal samples with calcein label (iOsx‐Ai9, *n* = 1; iDmp1‐Ai9, *n* = 2). Calcein^‐^ Surfaces: Four samples were removed from iOsx‐Ai9 ‐10 N female group, one sample removed from iDmp1‐Ai9 ‐10 N male group, and one sample removed from iDmp1‐Ai9 ‐7 N female group due to absence of calcein negative surface.

**Figure S5**. Percentage of TdT^+^ cells that are on surfaces adjacent to (i.e., overlying) calcein labels (as percent of total TdT^+^ cells on bone surface). Samples were excluded if no calcein was present, 100% of the surface was calcein positive, or if no TdT+ cells were on the surface; groups with <3 samples are not shown. *p<0.05 by unpaired t‐test.

**Figure S6** Number of proliferative cells per bone surface induced by loading. (A‐D) Total number of bone surface cells that proliferated or arose via proliferation (EdU^+^) in iOsx‐Ai9 (A) males and (B) females, and iDmp1‐Ai9 (C) males and (D) females. (E‐H) Number of cells on the bone surface that proliferated or arose via proliferation from a lineage positive cell (TdT^+^EdU^+^) in iOsx‐Ai9 (E) males and (F) females, and iDmp1‐Ai9 (G) males and (H) females. *p < 0.05, **p < 0.01, ***p < 0.001, ****p < 0.0001 by two‐way ANOVA repeated measures, Sidak multiple comparisons correction (factors: loading, force).

**Figure S7** Percentage of lineage positive cells on the bone surface that arose via proliferation. Percentage of TdT^+^ cells on the bone surface that were co‐labeled with EdU in iOsx‐Ai9 (A) males and (B) females, and iDmp1‐Ai9 (C) males and (D) females. *p < 0.05, **p < 0.01, ***p < 0.001, ****p < 0.0001 by two‐way ANOVA repeated measures, Sidak multiple comparisons correction (factors: loading, force).

**Table S1** Full list of standard dynamic histomorphometry measures from the periosteal surface.

**Table S2** Co‐localization of TdT^+^ cells and calcein label was similar to TdT^+^ percent along entire bone surface of loaded limbs. Percent values are computed from ratios of number of cells with relevant labels per total number of cells adjacent to calcein‐labeled (Calcein^+^) or non‐calcein‐labeled surfaces (Calcein^‐^) or per the total number of cells on the bone surface (BS).
