# Supplementary figures and images for: Dmp1 Lineage Cells Contribute Significantly to Periosteal Lamellar Bone Formation Induced by Mechanical Loading But Are Depleted from the Bone Surface During Rapid Bone Formation
Source: JBMR Plus. 2022 Jan 4;6(3):e10593. doi: 10.1002/jbm4.10593 (PMC8914163; doi:10.1002/jbm4.10593)

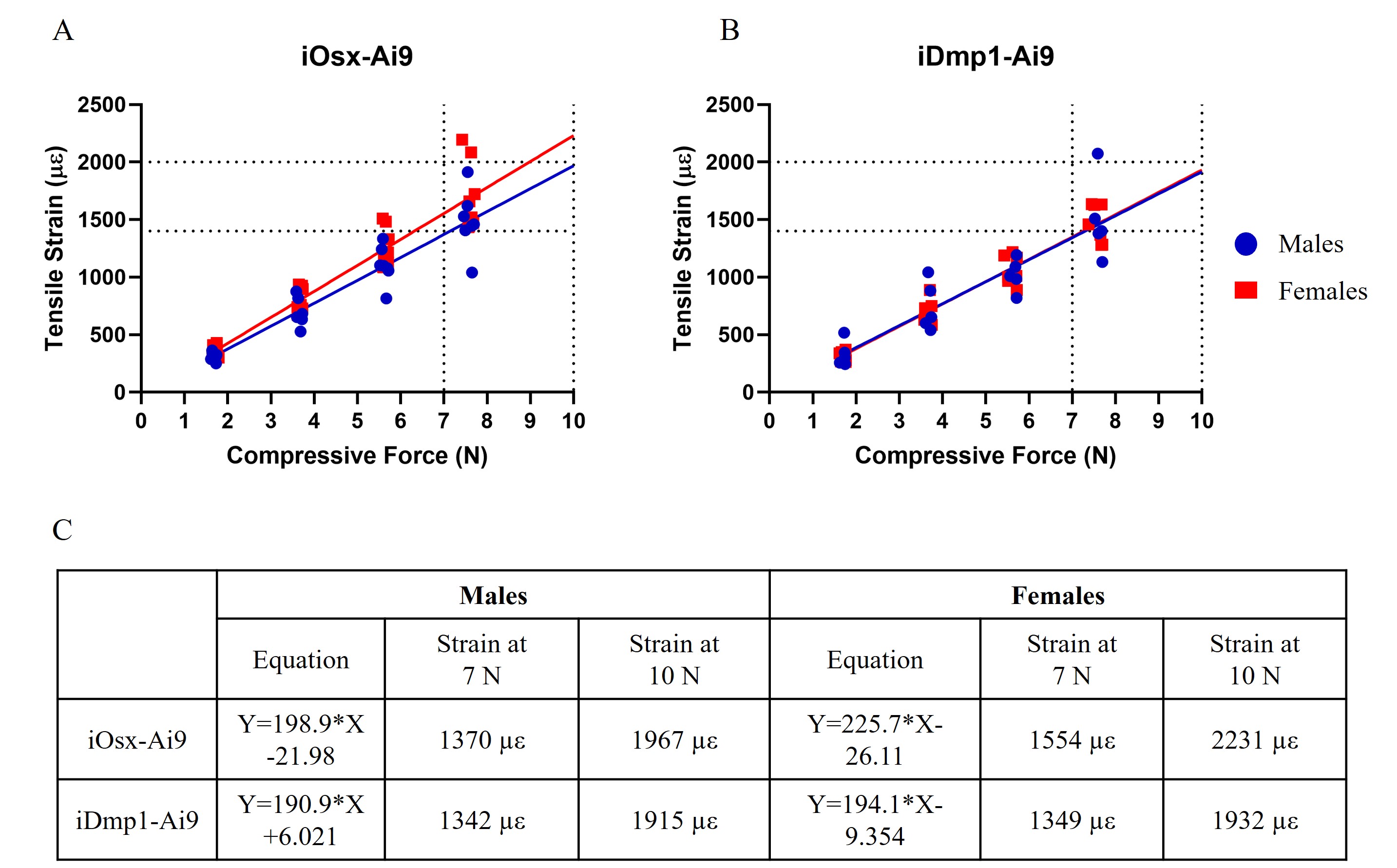

Supplement: Supplementary file 1 — Figure S1 Similar strains were engendered by 7 N and 10 N peak compressive forces across mouse strain and sex. Force‐strain relationship for (A) iOsx‐Ai9 (males n = 7; females n = 8) and (B) iDmp1‐Ai9 with respective linear regression lines are shown (males n = 5; females n = 7). (C) Linear regression equations for each group and the strains produced by 7 N and 10 N compressive forces. Higher strains (+180–260 με; +13%) were induced by these forces in female iOsx‐Ai9 mice compared to males; whereas the slope of the corresponding linear regression lines were not different, the y‐intercepts were significantly different (p = 0.0099). [file JBM4-6-e10593-s006.jpg]

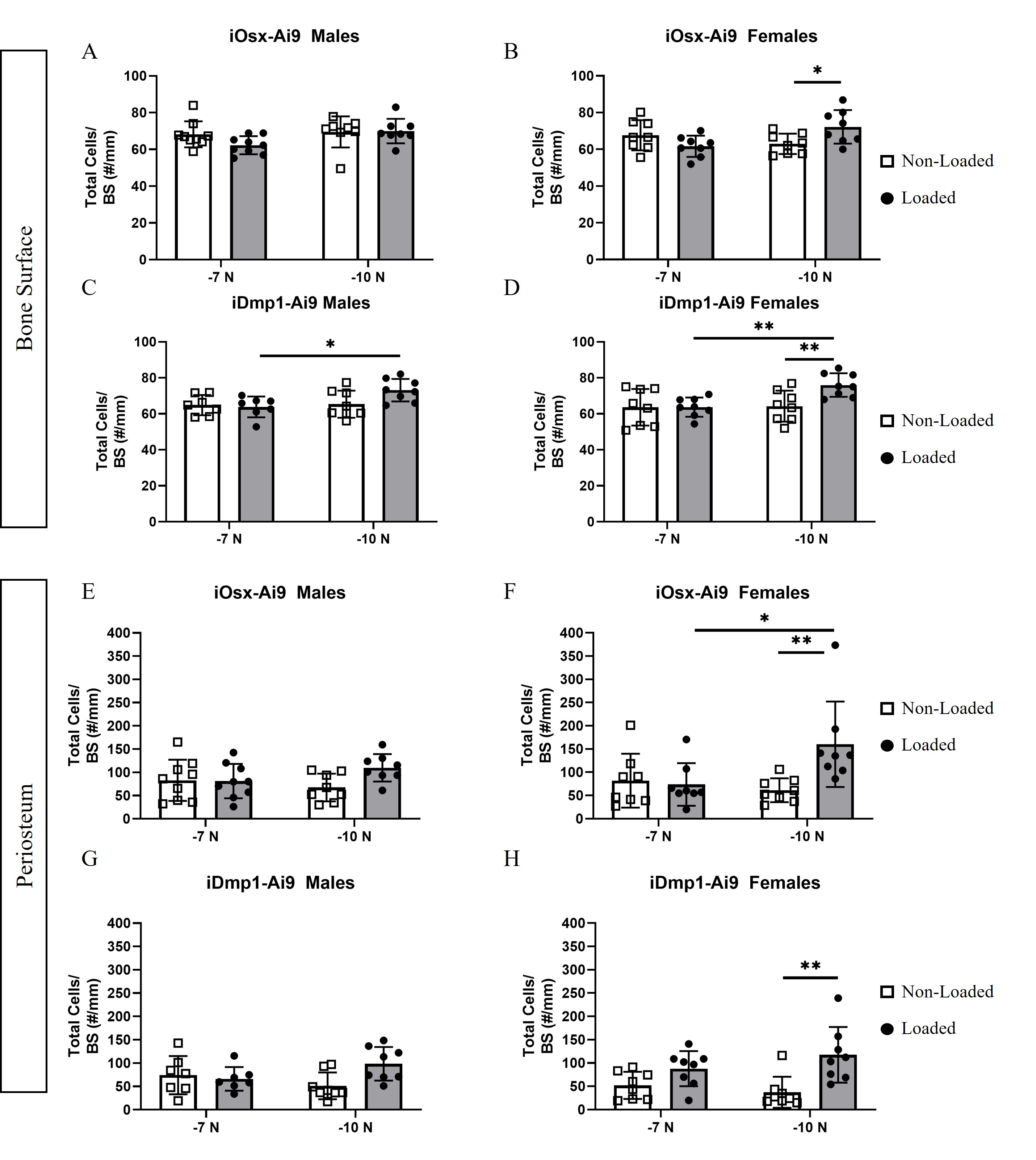

Supplement: Supplementary file 2 — Figure S2 Loading induced an increase in cell number at the bone surface and periosteum. (A‐D) Quantification of total number of cells at the bone surface normalized to bone surface length in iOsx‐Ai9 (A) males and (B) females and iDmp1‐Ai9 (C) males and (D) females. (E‐H) Quantification of total number of cells within the periosteum (not directly adjacent to the bone surface) normalized to bone surface length in iOsx‐Ai9 (E) males and (F) females and iDmp1‐Ai9 (G) males and (H) females. *p < 0.05, **p < 0.01, ***p < 0.001, ****p < 0.0001 by two‐way ANOVA repeated measures, Sidak multiple comparisons correction (factors: loading, force). [file JBM4-6-e10593-s003.jpg]

## No Tamoxifen Controls

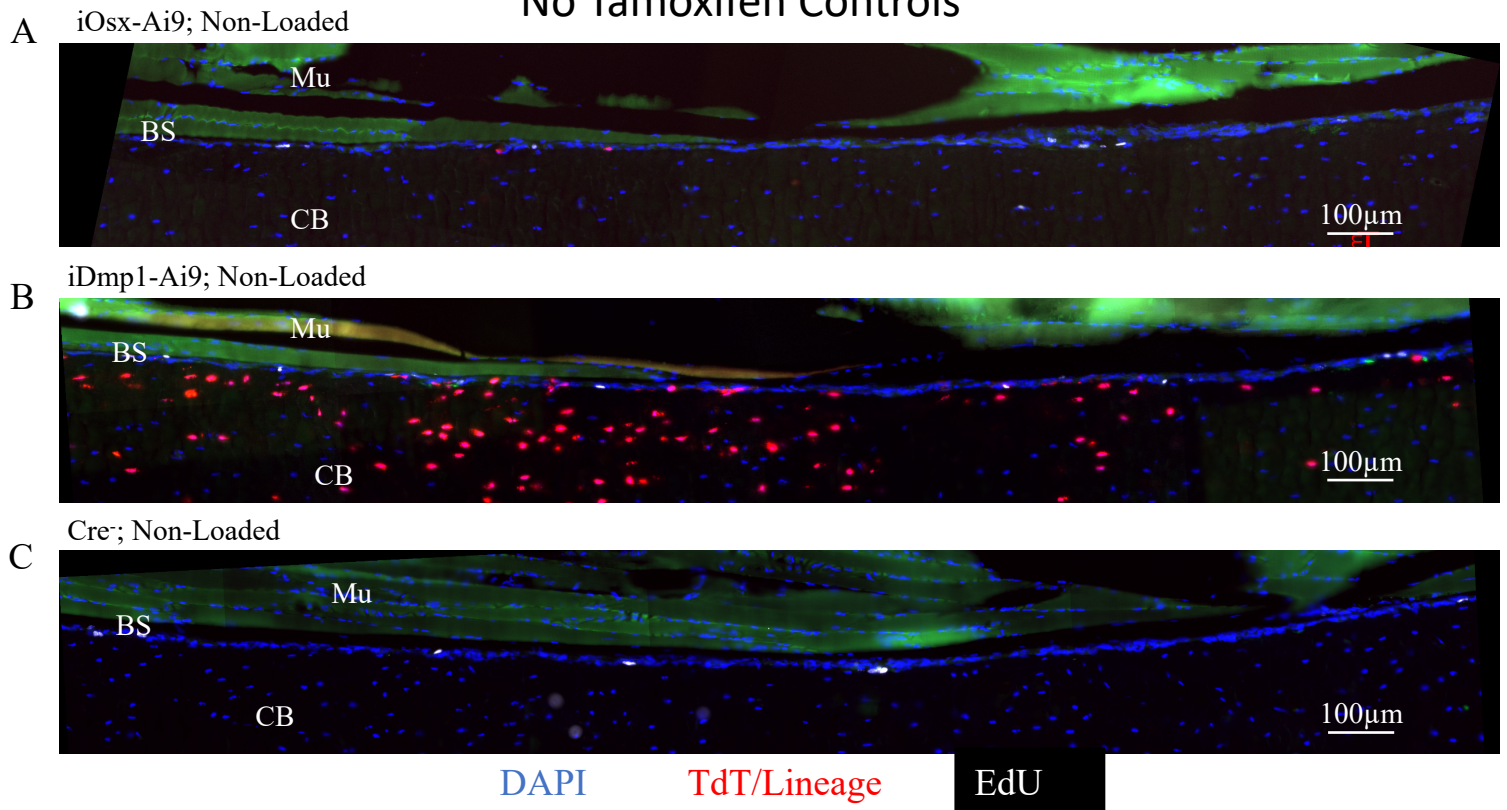

**D** iOsx-Ai9 No TAM

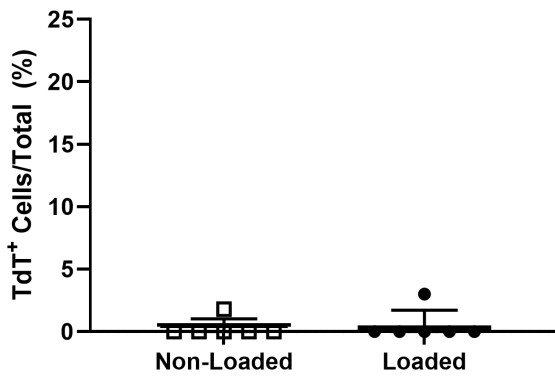

**E** iDmp1-Ai9 No TAM

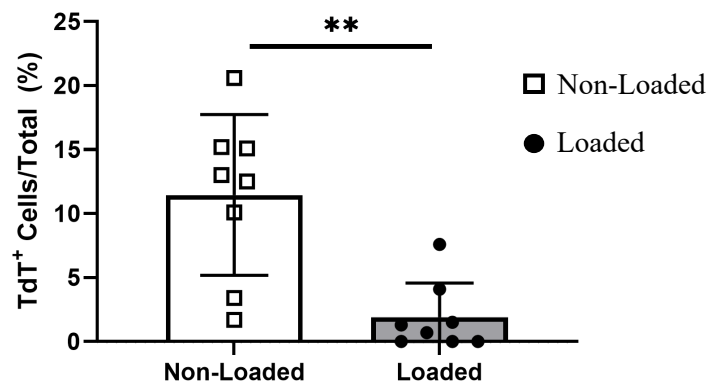

iDmp1-Ai9 Cortical Bone

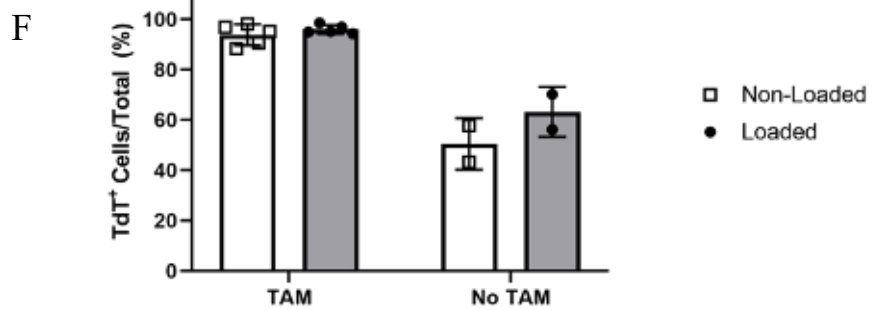

Supplement: Supplementary file 3 — Figure S3 TdTomato leakiness in iOsx‐Ai9 and iDmp1‐Ai9 mice not treated with tamoxifen. (A,B) Representative images of leakiness of (A) iOsx‐Ai9 and (B) iDmp1‐Ai9 mice without tamoxifen administration in non‐loaded limbs. (C) Representative image of a Cre− mouse where no TdT+ cells were found on the bone surface. (D,E) Quantification of the number of TdT+ cells on the bone surface normalized to total number of bone surface cells in (D) iOsx‐Ai9 and (E) iDmp1‐Ai9 mice that did not receive tamoxifen. (F) Percent TdT+ cells within the cortical bone of iDmp1‐Ai9 mice. **p < 0.01 by paired t test. Male and female data combined. No tamoxifen: iOsx‐Ai9 total n = 6 (males n = 3, females n = 3); iDmp1‐Ai9 total n = 8 (males n = 4, females n = 4). Cre Negative: iOsx‐Ai9 total n = 6 (males n = 3, females n = 3); iDmp1‐Ai9 total n = 7 (males n = 4, females n = 3). [file JBM4-6-e10593-s004.pdf]

A

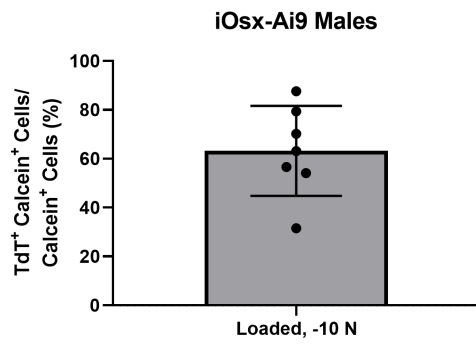

B

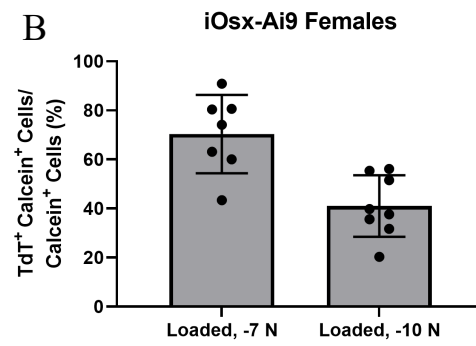

C

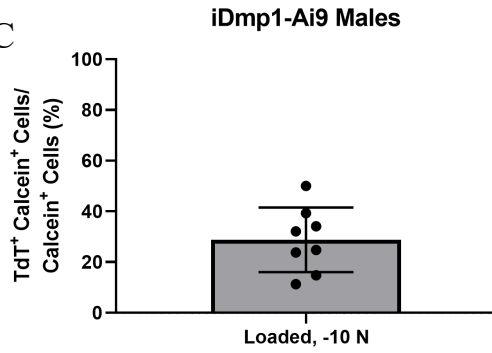

D

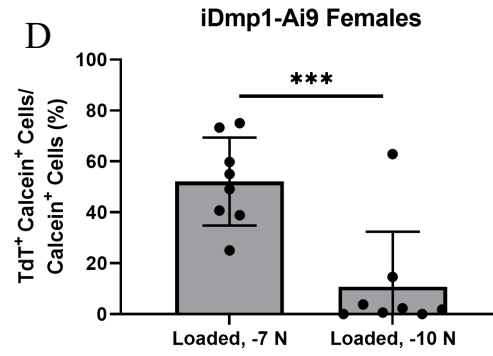

E

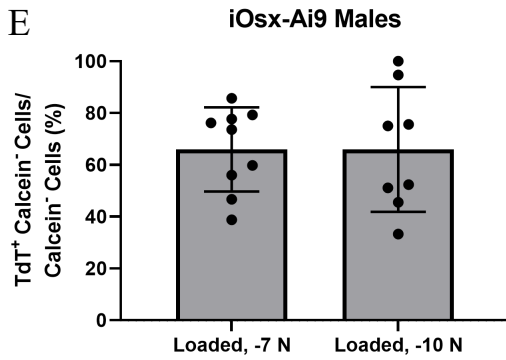

F

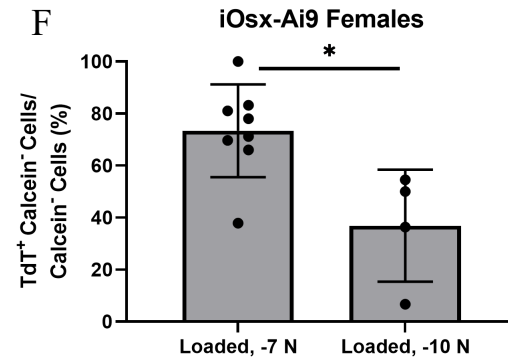

G

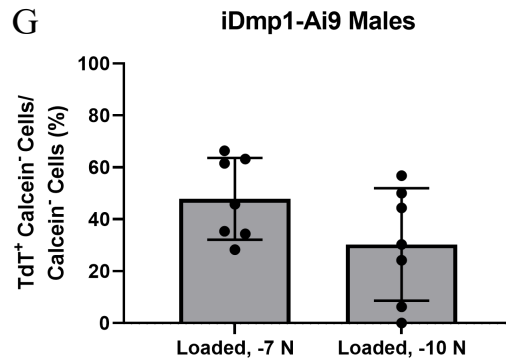

H

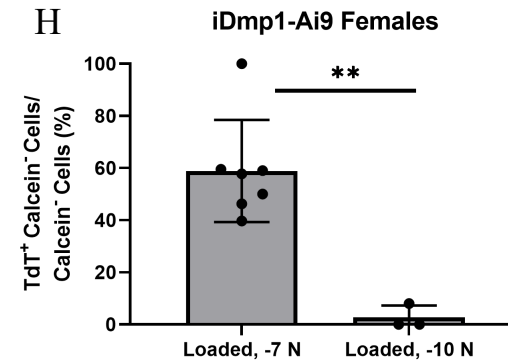

Supplement: Supplementary file 4 — Figure S4 Contribution of TdT+ cells to bone formation was similar at sites with or without calcein label. Percent values are computed from ratios of number of cells with TdT expression adjacent to (i.e., overlying) calcein label (TdT+Calcein+ Cells) per total number of cells adjacent to calcein label (Calcein+ Cells), or TdT+ cells not adjacent to calcein label (TdT+Calcein‐ Cells), per total cells not adjacent to calcein label (Calcein‐Cells). (A‐D) Quantification of TdT+ cells adjacent to calcein+ surfaces in loaded limbs of iOsx‐Ai9 (A) males and (B) females, and iDmp1 (C) males and (D) females.(E‐H) Quantification of TdT+ cells adjacent to calcein‐ surfaces in loaded limbs of iOsx‐Ai9 (E) males and (F) females, and iDmp1 (G) males and (H) females. *p < 0.05, **p < 0.01, ***p < 0.001, ****p < 0.0001 by unpaired t test. Calcein+ Surfaces: One sample was removed from iOsx‐Ai9 –10 N male group and one sample removed from iOsx‐Ai9 –7 N female group due to no presence of calcein. Male −7 N groups not included due to minimal samples with calcein label (iOsx‐Ai9, n = 1; iDmp1‐Ai9, n = 2). Calcein‐ Surfaces: Four samples were removed from iOsx‐Ai9 ‐10 N female group, one sample removed from iDmp1‐Ai9 ‐10 N male group, and one sample removed from iDmp1‐Ai9 ‐7 N female group due to absence of calcein negative surface. [file JBM4-6-e10593-s007.pdf]

A

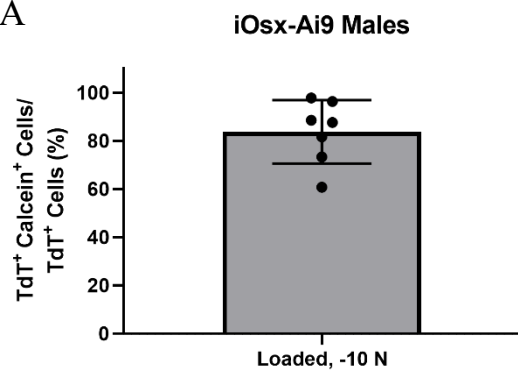

B

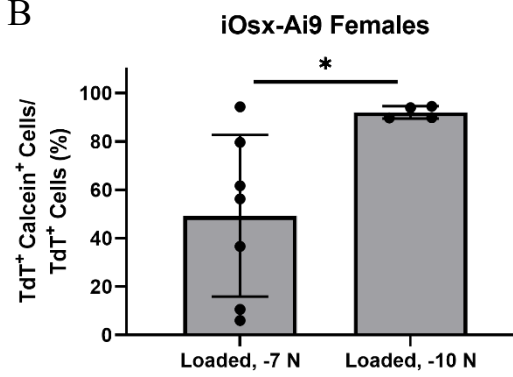

C

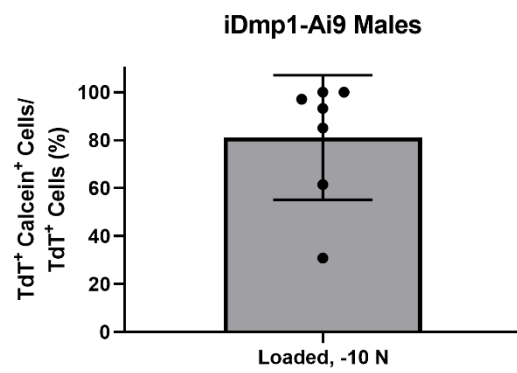

D

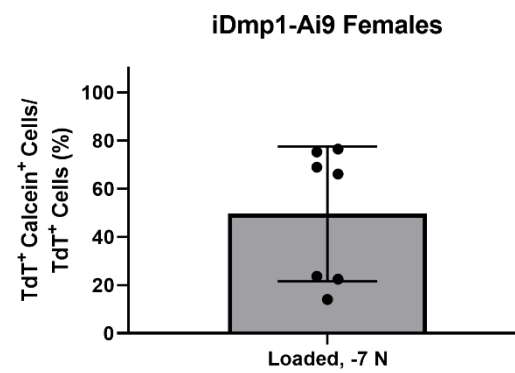

Supplement: Supplementary file 5 — Figure S5. Percentage of TdT+ cells that are on surfaces adjacent to (i.e., overlying) calcein labels (as percent of total TdT+ cells on bone surface). Samples were excluded if no calcein was present, 100% of the surface was calcein positive, or if no TdT+ cells were on the surface; groups with <3 samples are not shown. *p<0.05 by unpaired t‐test. [file JBM4-6-e10593-s008.pdf]

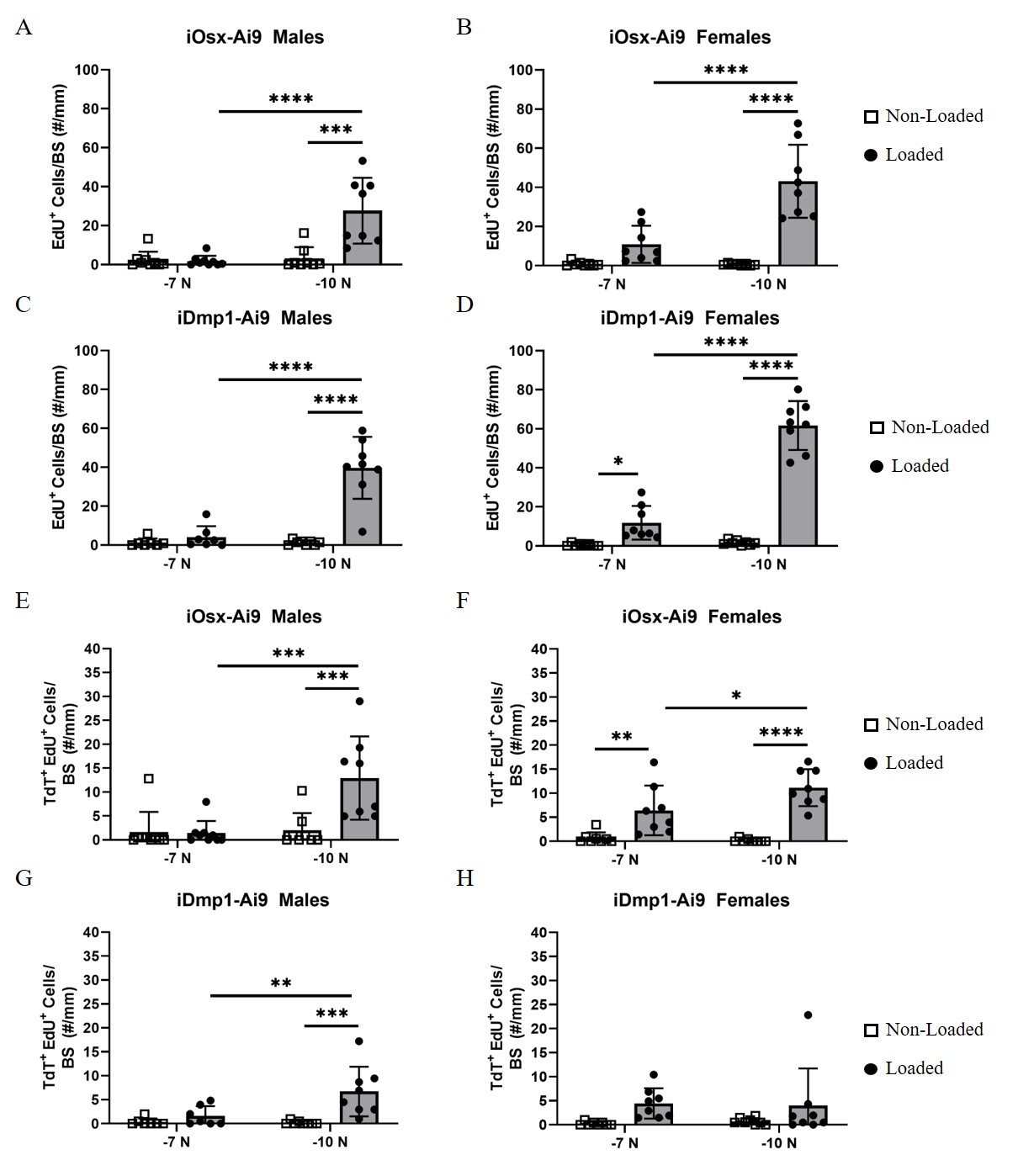

Supplement: Supplementary file 6 — Figure S6 Number of proliferative cells per bone surface induced by loading. (A‐D) Total number of bone surface cells that proliferated or arose via proliferation (EdU+) in iOsx‐Ai9 (A) males and (B) females, and iDmp1‐Ai9 (C) males and (D) females. (E‐H) Number of cells on the bone surface that proliferated or arose via proliferation from a lineage positive cell (TdT+EdU+) in iOsx‐Ai9 (E) males and (F) females, and iDmp1‐Ai9 (G) males and (H) females. *p < 0.05, **p < 0.01, ***p < 0.001, ****p < 0.0001 by two‐way ANOVA repeated measures, Sidak multiple comparisons correction (factors: loading, force). [file JBM4-6-e10593-s009.jpg]

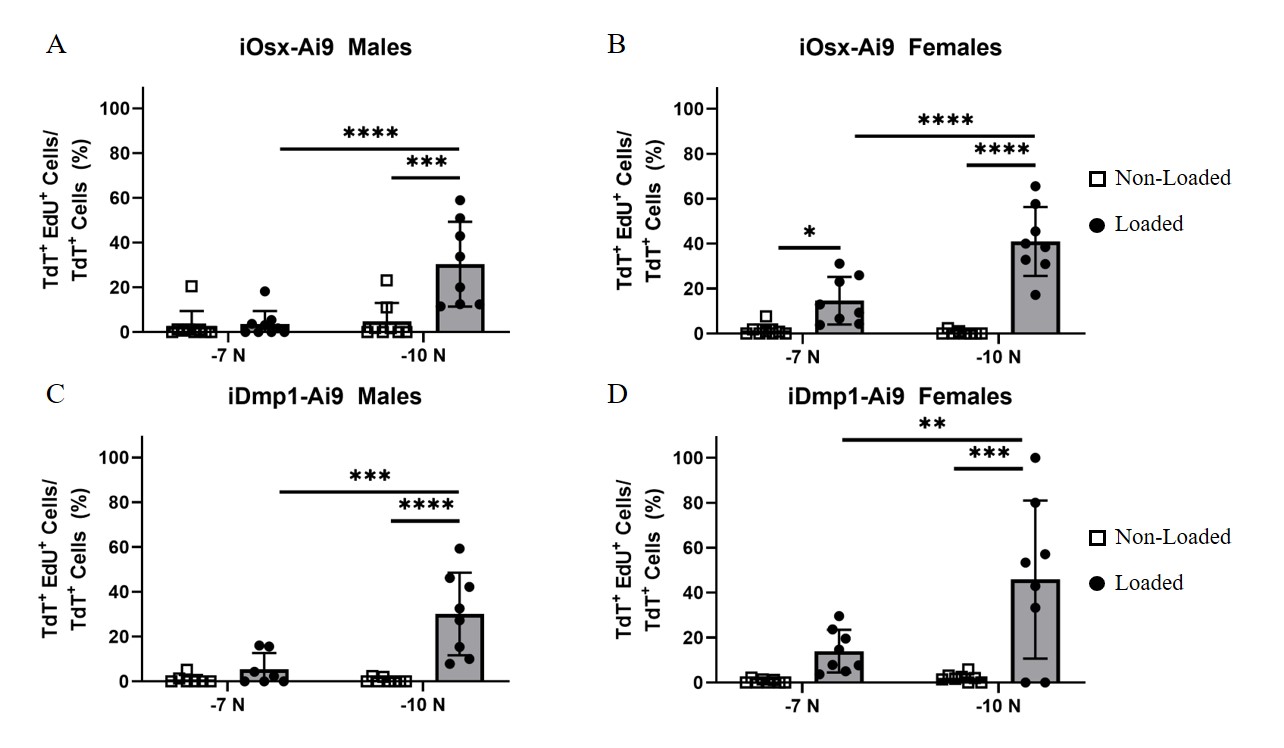

Supplement: Supplementary file 7 — Figure S7 Percentage of lineage positive cells on the bone surface that arose via proliferation. Percentage of TdT+ cells on the bone surface that were co‐labeled with EdU in iOsx‐Ai9 (A) males and (B) females, and iDmp1‐Ai9 (C) males and (D) females. *p < 0.05, **p < 0.01, ***p < 0.001, ****p < 0.0001 by two‐way ANOVA repeated measures, Sidak multiple comparisons correction (factors: loading, force). [file JBM4-6-e10593-s005.jpg]
